# Association of per- and polyfluoroalkyl substances (PFAS) with periodontitis: the mediating role of sex hormones

**DOI:** 10.1186/s12903-024-03863-0

**Published:** 2024-02-15

**Authors:** Yuxuan Wu, Yu Qiu, Yuying Wu, Husheng Li, Han Yang, Qingrong Deng, Baochang He, Fuhua Yan, Yanfen Li, Fa Chen

**Affiliations:** 1 Department of Epidemiology and Health Statistics, School of Public Health, Fujian Medical University, Fuzhou, 350122 China; 2 Department of Oral and Maxillofacial Surgery, The First Affiliated Hospital of Fujian Medical University, Fuzhou, 350122 China; 3 Department of Preventive Medicine, School of Public Health, Fujian Medical University, Fuzhou, 350122 China; 4 Department of Periodontology, Nanjing Stomatological Hospital, Medical School of Nanjing University, Nanjing, 210008 China

**Keywords:** Periodontitis, PFAS, PFOS, PFNA, Population-based study, NHANES

## Abstract

**Objectives:**

To investigate the association between serum per- and polyfluoroalkyl substances (PFAS) and periodontitis, and further explore the possible mediating role of sex hormones in this association.

**Methods:**

We extracted data from National Health and Nutrition Examination Survey (NHANES) 2009–2014. Univariable and multivariable logistic regression models were performed to investigate the association between serum levels of seven PFASs and periodontitis. Bayesian kernel machine regression (BKMR) was conducted to assess the joint effect of PFASs in mixtures. Mediation analyses were used to explore the potential mediating role of sex hormones.

**Results:**

Participants with periodontitis had higher concentrations of serum perfluorooctane sulfonate (PFOS) and perfluorononanoic acid (PFNA) than those without periodontitis (both *P* < 0.05). In fully adjusted models, high serum concentrations of PFOS and PFNA were positively associated with periodontitis (tertile 3 vs. tertile 1: prevalence ratio (PR) = 1.19 for PFOS, 95% CI: 1.01–1.39; PR = 1.17 for PFNA, 95% CI: 1.02–1.34). The results from the BKMR models consistently showed a positive association between PFAS mixtures and periodontitis. Of note, testosterone and the ratio of testosterone to estradiol significantly mediated the relationship between high level of PFOS and periodontitis, accounting for 16.5% and 31.7% of the total effect, respectively. Sensitivity analyses yielded similar results when using periodontal clinical indices (mean loss of attachment, mean periodontal probing depth, and the number of teeth) as dependent variables.

**Conclusions:**

These findings provide evidence to support a positive association between certain PFASs and periodontitis, which might be partially mediated by sex hormones.

**Supplementary Information:**

The online version contains supplementary material available at 10.1186/s12903-024-03863-0.

## Introduction

Periodontitis, a common chronic inflammatory disease, is characterized by gingival inflammation, periodontal pocket formation, and gradual destruction of tooth-supporting tissues, which is a major cause of tooth loss and seriously affects oral and systemic health. Over the past 30 years, the prevalence of periodontitis has markedly increased worldwide, with the number affected increasing from 0.5 billion to nearly 1.1 billion [1]. A continual rise over the next several years is anticipated due to rapid population aging [2]. Therefore, identifying risk factors for periodontitis is crucial to understand its etiology and develop corresponding preventive strategies.

Most studies concerning the risk factors for periodontitis have focused on periodontal pathogens, lifestyles, and genetic factors [3]. In the past few years, the effects of environmental factors on periodontitis have received increasing attention. Per- and polyfluoroalkyl substances (PFAS) are new persistent organic pollutants (POPs) that have become ubiquitous in the environment. Owing to their hydrophobicity, oleophobicity, and thermal stability, PFASs have been extensively applied in multifarious commercial products and industrial applications for the past few decades, such as heat- and oil-tolerant food packaging, nonstick pan coatings, firefighting foams, and surfactants [4]. Dietary intake (such as seafood, meat and meat products, and dairy products), drinking water (particularly that near contaminated areas), and contact with other contaminated media (such as carpets, paper, and food packaging materials) are the major sources of human exposure to various PFASs [5]. A number of PFASs were detected in serum samples from more than 98% of the U.S. population [6]. Accumulating evidence indicates that PFASs have toxic effects on various biological systems, including the endocrine [7], reproductive [8], and immune systems [9].

To date, several epidemiological studies have reported that PFAS exposure is associated with human health outcomes, such as metabolic syndrome [10], cardiovascular events [11], and cancer [12]. A growing number of studies have revealed that PFASs can disrupt sex hormones and affect bone cell differentiation, ultimately resulting in enhanced bone resorption [13–16], which is similar to the important features of periodontitis. However, no population-based studies have explored the relationship between PFAS exposure and periodontitis. Therefore, using data from the National Health and Nutrition Examination Survey (NHANES) 2009–2014, we investigated the association between serum PFAS and periodontitis and further explored whether sex hormones mediated this association.

## Methods

### Study population

The data of this cross-sectional study were extracted from the NHANES 2009–2014. This survey involves annual assessments of a nationally representative sample of approximately 5,000 persons. These individuals are distributed in counties across the country, with 15 of counties are visited each year. This span included three 2-year cycles; these 6 years were the only years during which the NHANES protocol included full-mouth periodontal examination, excluding the third molars.

**Fig. 1:**
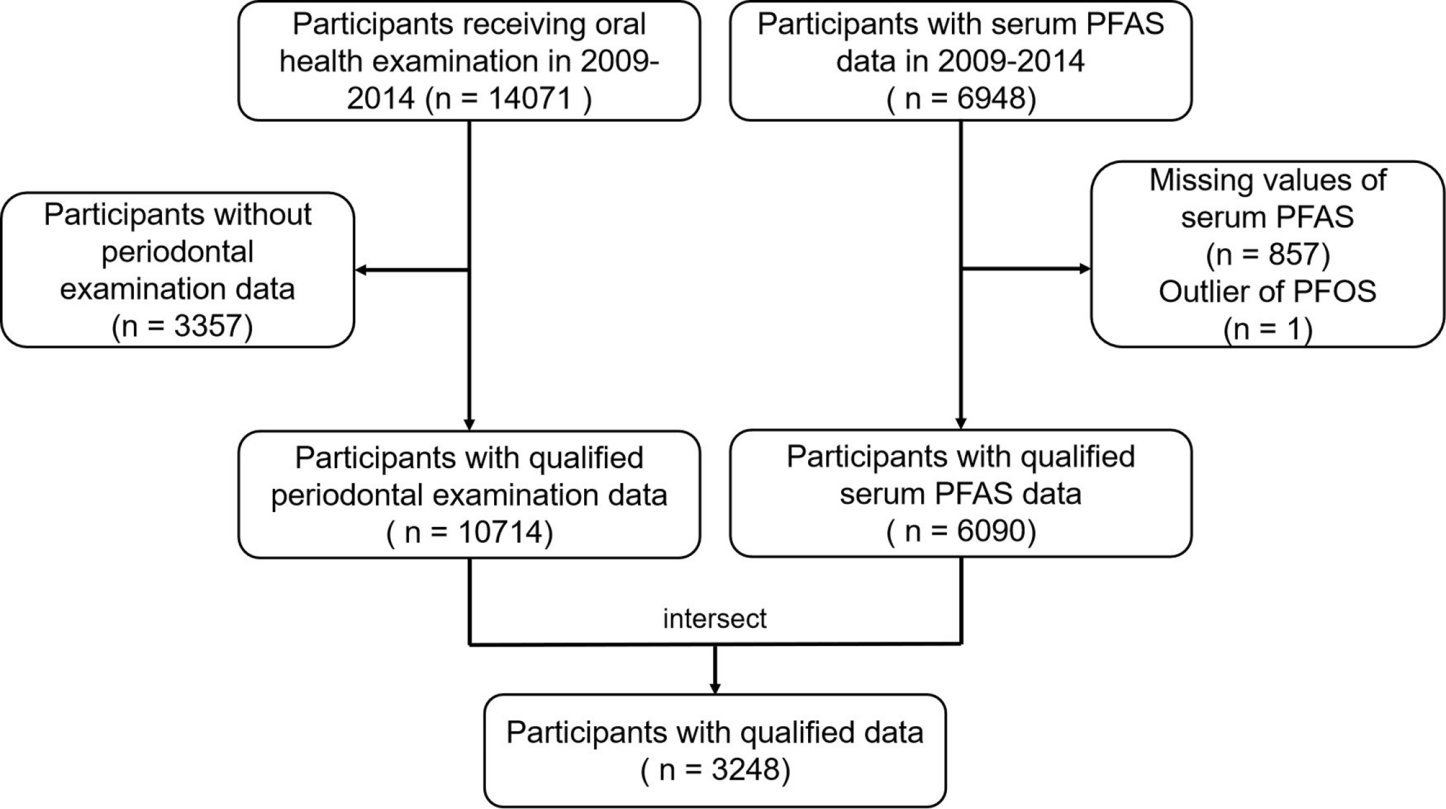
Flowchart of study in NHANES 2009–2014 (*N* = 3248)

After excluding individuals with incomplete periodontal examination (*n* = 3357) and serum PFAS level data (*n* = 857), as well as an outlier of serum PFOS levels (1403 ng/ml) that deviated significantly from the norm for the general population or residents in PFAS-contaminated areas [17, 18], a total of 3248 participants were ultimately included in the final analysis. Further details can be found in Fig. 1. Furthermore, a post-hoc calculation of statistical power was conducted using the software (PASS 2021). With a significance level (α) set at 0.05, a prevalence ratio (PR) of 1.24 (range 1.00 to 1.47), an exposure proportion of the control group at 0.31 (range 0.26 to 0.38), the calculated post-hoc statistical power was found to be 80.98%. These findings indicate that the sample size for the study was sufficient.

On the dedicated website (www.cdc.gov/nchs/nhanes.htm), information is available on the technical aspects of the project, such as the sample design, periodontal data collection techniques, and data availability. The oral health data collection protocols were approved by the Ethics Review Board of the National Center for Health Statistics (NCHS) of the Centers for Disease Control and Prevention (CDC), and all survey participants signed an informed consent form. This study was conducted in accordance with the Declaration of Helsinki (1975; revised in 2013) and was exempt from the need for approval from the Institutional Review Board of Fujian Medical University because we used deidentified information from the NHANES database. We have complied with the STROBE protocol.

### Periodontal examination

Participants aged between 30 and 80 years were eligible for periodontal examination in the NHANES 2009–2014. Using the HU-Friedy perioprobe, which is color-coded and marked at increments of 2 mm, gingival recession and periodontal probing depth (PPD) were measured at six sites per tooth (the disto-facial, mid-facial, mesio-facial, disto-lingual, mid-lingual, and mesio-lingual sites). The data input program calculated the loss of attachment (AL) (difference between PPD and gingival recession) [19].

Participants who completed a periodontal examination were categorized according to the CDC-AAP case criteria for periodontitis [20]. Mild periodontitis was classified as having ≥ 2 sites with AL ≥ 3 mm and ≥ 2 sites with PPD ≥ 4 mm (not on the same tooth) or 1 site with PPD ≥ 5 mm. Moderate periodontitis was defined as having ≥ 2 interproximal sites with AL ≥ 4 mm (not on the same tooth) or ≥ 2 interproximal sites with PPD ≥ 5 mm (not on the same tooth). Severe periodontitis was defined as having ≥ 2 interproximal sites with AL ≥ 6 mm (not on the same tooth) and ≥ 1 interproximal site with PPD ≥ 5 mm. The absence of any of the above features of periodontitis was defined as periodontitis free [20]. In our investigation, periodontitis status was dichotomized into two categories (yes/no). “Yes” encompassed mild, moderate, or severe periodontitis [21].

### Measurement of PFAS levels

The NHANES used tandem mass spectrometry to detect and quantify serum levels of PFASs [22]. For analytes with levels below the lowest limit of detection (LLOD, in ng/mL), the results were computed using LLOD/√2. The LLOD for each poly-fluorochemical included in our study is shown in Appendix Table 1. The NHANES Laboratory Procedures Manual contains more information about analytical methods and procedures. We selected seven PFASs that were measured in the NHANES 2009–2014: perfluorooctanoic acid (PFOA), perfluorooctane sulfonic acid (PFOS), perfluorohexane sulfonate acid (PFHxS), 2-(N-methyl-perfluorooctane sulfonamido) acetic acid (MPAH), perfluoroundecanoic acid (PFUA), perfluorononanoic acid (PFNA), and perfluorodecanoic acid (PFDE).

### Covariates

We constructed a Directed Acyclic Graph (DAG) to assess potential confounding variables (Appendix Fig. 1). Demographic variables in our study included age, sex, race/ethnicity, educational attainment, marital status, and the poverty income ratio (PIR). For our study, senior citizens were defined as those aged 65 years or older. The PIR was calculated by dividing the family income by the poverty level [23]. Other covariates included smoking status, alcohol intake (drinks per day), body mass index (BMI), hypertension, and diabetes. Participants were classified as “nonsmokers” (smoked less than 100 cigarettes in their lifetime), “former smokers” (had smoked in the past but had quit before the time of the interview), or “current smokers” (smoked at least 100 cigarettes throughout their lifetime and continued to smoke at the time of the interview) [24]. BMI was calculated by dividing the individual’s weight in kilograms by the square of his height in meters (kg/m^2^). According to the World Health Organization, overweight was classified as BMI ≥ 25 kg/m^2^ and obesity was classified as BMI ≥ 30 kg/m^2^. The present study employed the multiple imputation algorithm to address missing covariate data. Specifically, the “mice” package in R was utilized to perform multiple interpolation, with the “polyreg” method applied to categorical variables and the “pmm” method applied to continuous variables [25]. The number of interpolations was set to five.

### Statistical analysis

Participants’ demographic characteristics and serum PFAS levels are presented as the number (proportion), mean (standard deviation), and median (25th to 75th percentiles) for categorical variables, normally distributed continuous variables, and nonnormally distributed continuous variables, respectively. We compared sociodemographic characteristics between the periodontitis and non-periodontitis groups using the chi-squared test or Mann‒Whitney U test, as appropriate. We used the chi-squared test or Kruskal-Wallis H test to compare the sociodemographic characteristics and PFAS levels of participants with different levels of periodontitis. Based on serum concentration tertiles, levels of the seven PFASs were also classified into three categories.

Considering the complex sampling of NHANES, we weighted the data using specific sample weights provided by the NHANES to ensure that these data were representative of the noninstitutionalized resident population in the US. According to weight selection guidelines, mobile examination center (MEC) weights of subsamples for PFAS detection were used in this study. Appendix Table 2 shows the code for the weight adjustment procedure.

To examine the association between serum PFAS levels and periodontitis, we performed robust Poisson regression analyses to calculate prevalence ratios (PRs) with 95% CIs [26]. PRs for all outcomes were adjusted for age, sex, race/ethnicity, educational attainment, marital status, BMI, smoking status, alcohol intake, poverty income ratio, serum cotinine and milk product consumption.

Restricted cubic splines (RCS) were utilized to determine the dose‒response relationship between PFAS levels and periodontitis. We also analyzed whether these seven PFASs had an effect on the risk of different levels of periodontitis using unordered multivariable logistic regression. Collinearity diagnostics on age, sex, race/ethnicity, educational attainment, marital status, BMI, smoking status, alcohol intake, the poverty income ratio, serum cotinine and milk product consumption were performed to prove no severe collinearity (both variance inflation factor (VIF) < 3, see Appendix Table 3). When VIF is > 10, it is considered to indicate a severe collinearity [27].

Due to the high correlations among the 7 PFASs, we analyzed the mixed effects of seven PFASs on periodontitis using the Bayesian kernel machine regression (BKMR) model [28]. To estimate individual PFAS contributions to the cumulative effect, we evaluated the health effects of exposure to any single PFAS, defined as a change in the specific PFAS from the 25th percentile to 75th percentile with all other PFASs fixed at a particular percentile (25th, 50th, and 75th) [29]. Mediation analyses were used to explore the mediation effects of sex hormones (testosterone, estradiol, sex hormone-binding globulin (SHBG) and TT/E_2_ (a ratio of testosterone to estradiol; an indirect assessment of circulating free testosterone)) on the relationship between PFAS levels and periodontitis. Since the NHANES 2009–2012 survey cycles did not provide specific information on sex hormones, we focused on NHANES 2013–2014 survey cycle with complete sex hormone data (*n* = 1055) for mediation analyses. We performed mediation analyses to evaluate the potential mediators in the association between the PFASs (three-category variables) and periodontitis. The Appendix Fig. 2 showed the schematic of a simple mediation model. Indirect effects are significantly defined as having a mediating effect [30]. We performed 5000 bootstrap mediations using the PROCESS V4.1 macros for SPSS (version 20.0) [31].

**Fig. 2:**
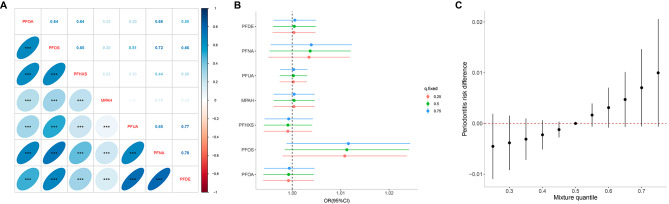
The associations of PFAS mixtures and single-PFAS with periodontitis in NHANES 2009–2014 (*N* = 3248). **A**: Heatmap of seven PFASs correlation; **B**: The overall effect of PFAS mixtures using the BKMR model; **C**: Association of single PFAS with periodontitis when other PFAS are fixed at a specific quantile (25th, 50th, and 75th). Adjusted by age, sex, race/ethnicity, educational attainment, marital status, BMI, smoking status, alcohol intake, milk product consumption, poverty income ratio, and serum cotinine

### Subgroup analysis and sensitivity analysis

In this study, an interaction term, created by multiplying PFAS and the stratifying variables, was added to the model, and statistical interactions were individually tested in each model using likelihood ratio tests. For sensitivity analysis, weighted linear regression was used to evaluate the relationship between PFAS levels and periodontal clinical indices (mean AL, mean PPD, and the number of teeth). Due to the skewed distribution of these indices, they were log-transformed to improve the normality of distribution for regression analysis. Furthermore, we further conducted an assessment of potential residual confounding using E-values. E-values were calculated to determine the minimum strength required for unmeasured confounders to eliminate the observed association between the exposure and outcome [32].

A two-tailed *P* < 0.05 was considered statistically significant. For all analyses, we used R (version 4.3.0).

## Results

### Demographic characteristics

As shown in Table 1, there were 1643 (41.2%) participants with periodontitis and 1605 (58.8%) participants without periodontitis. Compared to those without periodontitis, participants with periodontitis were more likely to be older adults (age ≥ 65 years), male, Mexican American or non-Hispanic Black individuals, former or current smokers. In addition, participants with periodontitis tended to have lower levels of education, a lower poverty income ratio, and higher alcohol consumption than those without periodontitis. Of the 1643 participants with periodontitis, 154 participants (9.4%) had mild periodontitis, 1157 participants (70.4%) had moderate periodontitis, and 332 participants (20.2%) had severe periodontitis, all covariates except BMI and milk product consumption were distributed differently across groups (*P* < 0.05).

**Table 1:** Demographic characteristics of the total population in NHANES 2009–2014 (*N* = 3248)

Characteristics	Total *N* = 3248	Participants with no periodontitis *n* = 1605 (58.8%)	Participants with periodontitis *n* = 1643 (41.2%)	*P* value
Continuous variables, mean (SD)				
Age (years)	50.79(0.31)	48.21(0.49)	54.48(0.42)	< 0.001
Alcohol intake (drinks/day)	2.47(0.06)	2.24(0.06)	2.80(0.10)	< 0.001
Poverty income ratio, PIR	3.14(0.05)	3.45(0.06)	2.71(0.06)	< 0.001
Serum Cotinine (ng/mL)	49.42(3.11)	33.30(2.97)	72.44(4.81)	< 0.001
Categorical variables, n (%)				
Age group				< 0.001
Youth and middle (Age ˂ 65)	2538(82.1)	1359(86.6)	1179(75.7)	
Old (Age ≥ 65)	710(17.9)	246(13.4)	464(24.3)	
Sex				< 0.001
Male	1599(49.2)	657(43.3)	942(57.5)	
Female	1649(50.8)	948(56.7)	701(42.5)	
BMI (kg/m^2^)				0.241
Normal	881(27.5)	454(28.8)	427(25.6)	
Overweight	1139(35.3)	559(35.2)	580(35.4)	
Obese	1228(37.3)	592(36.0)	636(39.1)	
Race/ethnicity				< 0.001
Non-Hispanic White	1418(69.1)	832(75.8)	586(59.6)	
Non-Hispanic Black	627(9.9)	237(7.2)	390(13.8)	
Mexican American	471(8.1)	156(5.0)	315(12.5)	
Other	732(12.8)	380(12.0)	352(14.1)	
Educational attainment				< 0.001
Less than High school	792(15.9)	229(9.2)	563(25.5)	
High school and college or above	2456(84.1)	1376(90.8)	1080(74.5)	
Marital status				< 0.001
Married	1945(64.2)	1035(69.4)	910(56.9)	
Unmarried but have/had partner	937(25.6)	382(20.9)	555(32.3)	
Never married	366(10.2)	188(9.7)	178(10.9)	
Smoking status				< 0.001
Never	1858(57.3)	1035(63.7)	823(48.1)	
Former	813(25.7)	368(24.6)	445(27.3)	
Current	577(17.0)	202(11.7)	375(24.6)	
Milk product consumption				0.349
Never	585(16.8)	279(16.4)	306(17.3)	
Rarely-less than once a week	508(14.9)	271(15.8)	237(13.7)	
Sometimes-once a week or more, but less than once a day	910(27.2)	456(27.7)	454(26.4)	
Often-once a day or more	1231(40.6)	592(39.5)	639(42.3)	
Varied	14(0.4)	7(0.5)	7(0.2)	

Note: Continuous variables are represented by mean (weighted standard deviation), categorical variables are represented by n (weighted %).There are 219 (6.7%) missing values in alcohol intake (drinks/day), 304 (9.4%) missing values in poverty income ratio, 25 (0.8%) missing values in BMI (kg/m^2^), 3 (0.09%) missing values in marital status, 2 (0.06%) missing values in smoking status, 6 (0.2%) missing values in educational attainment, 1 missing value (0.03%) in serum cotinine (ng/mL). Missing data on covariates were processed using the multiple imputation algorithm

### Serum PFAS levels in different participants

The detection rates of seven PFASs in serum are shown in Appendix Table 1. The detection rates were in the range of 59.0–99.7%. As presented in Appendix Fig. 3 and Appendix Table 4, participants with periodontitis, compared with their counterparts without periodontitis, had higher serum concentrations of PFOS and PFNA (both *P* < 0.05). PFOA, PFHxS, MPAH, PFUA, and PFDE levels were higher in participants with periodontitis but did not reach statistical significance. Additionally, a statistically significant disparity in PFOS levels (both continuous and categorical) was observed among participants with varying degrees of periodontitis severity, as presented in Appendix Table 5 (*P* < 0.05).

**Fig. 3:**
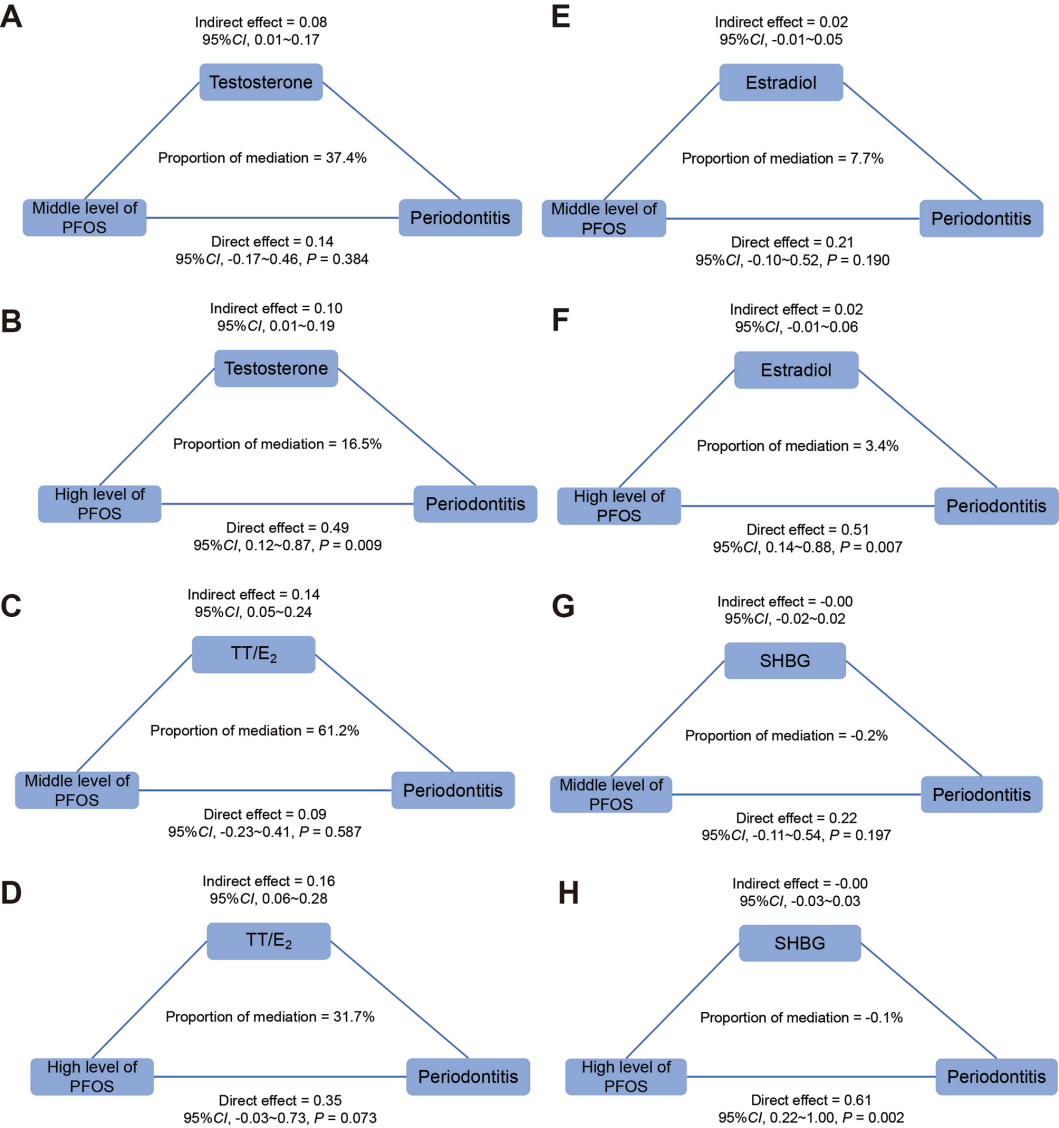
Mediation analyses of the association between PFOS and periodontitis by (**A**, **B**) Testosterone (**C**, **D**) TT/E2 (a ratio of testosterone to estradiol) (**E**, **F**) Estradiol (**G**, **H**) SHBG (sex hormone binding protein). Adjusted by age, race/ethnicity, educational attainment, marital status, BMI, smoking status, alcohol intake, milk product consumption, poverty income ratio, and serum cotinine

### Association between serum PFAS levels and periodontitis

The results from univariable logistic regression showed that high serum concentrations of PFOS and PFNA were positively related to periodontitis (Table 2). After adjusting for confounding factors, high serum concentrations of PFOS (PR = 1.19, 95% CI: 1.01–1.39; *P* = 0.033) and PFNA (PR = 1.17, 95% CI: 1.02–1.34; *P* = 0.024) were significantly associated with periodontitis compared with lower serum concentrations. The participants were further divided into non-periodontitis, mild, moderate, and severe periodontitis groups, the characteristics of participants with different periodontal conditions were shown in Appendix Table 6. As illustrated in Appendix Table 7, elevated concentrations of PFOA, PFOS, MPAH, PFNA, and PFDE were associated with mild periodontitis (*P* < 0.05). Furthermore, heightened levels of PFOS, PFUA, PFNA, and PFDE were linked to moderate periodontitis (*P* < 0.05), while increased levels of PFOS, PFNA, and PFDE were significantly correlated with severe periodontitis (*P* < 0.05). RCS analysis revealed a dose-response relationship between the levels of PFOS or PFNA and periodontitis (Appendix Fig. 4). The risk of periodontitis was found to increase with higher doses of PFOS and PFNA.

**Table 2:** PR (95%CI) for PFAS and periodontitis risk in the NHANES from 2009–2014 (*N* = 3248)

Categories	Unadjusted model		Adjusted model	
	PR (95%CI)	*P* value	PR (95%CI)	*P* value
PFOA [tertile (ng/mL)]				
Low (<1.87)	Ref	Ref	Ref	Ref
Middle (1.87–3.17)	1.00(0.84,1.19)	0.998	0.96(0.87,1.06)	0.429
High (≥ 3.17)	0.95(0.76,1.20)	0.685	0.97(0.86,1.08)	0.546
PFOS [tertile (ng/mL)]				
Low (<5.40)	Ref	Ref	Ref	Ref
Middle (5.40–10.90)	1.07(0.81,1.41)	0.646	1.05(0.89,1.24)	0.569
High (≥ 10.90)	1.47(1.13,1.91)	**0.005**	1.19(1.01,1.39)	**0.033**
PFHxS [tertile (ng/mL)]				
Low (<1.00)	Ref	Ref	Ref	Ref
Middle (1.00-2.07)	1.22(1.02,1.46)	**0.031**	1.00(0.90,1.11)	0.995
High (≥ 2.07)	1.12(0.88,1.43)	0.346	0.92(0.80,1.06)	0.259
MPAH [tertile (ng/mL)]				
Low (<0.07)	Ref	Ref	Ref	Ref
Middle (0.07–0.20)	0.97(0.79,1.20)	0.771	0.96(0.85,1.08)	0.499
High (≥ 0.20)	1.25(0.99,1.58)	0.061	1.09(0.95,1.25)	0.199
PFUA [tertile (ng/mL)]				
Low (<0.07)	Ref	Ref	Ref	Ref
Middle (0.07–0.20)	0.93(0.71,1.23)	0.620	1.02(0.87,1.19)	0.829
High (≥ 0.20)	1.12(0.85,1.48)	0.417	1.15(0.99,1.33)	0.060
PFNA [tertile (ng/mL)]				
Low (<0.74)	Ref	Ref	Ref	Ref
Middle (0.74–1.30)	1.14(0.92,1.42)	0.220	1.05(0.92,1.19)	0.459
High (≥ 1.30)	1.36(1.05,1.77)	**0.022**	1.17(1.02,1.34)	**0.024**
PFDE [tertile (ng/mL)]				
Low (<0.20)	Ref	Ref	Ref	Ref
Middle (0.20–0.30)	1.06(0.84,1.35)	0.608	1.08(0.93,1.24)	0.310
High (≥ 0.30)	1.07(0.81,1.40)	0.634	1.08(0.94,1.24)	0.287

Note: Ref = Reference. Adjusted by age, sex, race/ethnicity, educational attainment, marital status, BMI, smoking status, alcohol intake, milk product consumption, poverty income ratio, and serum cotinine

Additionally, there was a high correlation among the seven PFASs (Fig. 2A). Using the Bayesian kernel machine regression (BKMR) model, PFOS was associated with higher odds of periodontitis when the other PFASs were fixed at their 25th, 50th, and 75th percentiles (Fig. 2B). Figure 2C depicts the overall effect of the seven PFAS mixtures on periodontitis, indicating a positive dose-response relationship.

### Mediating role of sex hormones

As shown in Fig. 3, we explored the mediation effects of sex hormones (testosterone, estradiol, SHBG, and TT/E_2_) on the relationship between PFOS and periodontitis in NHANES 2013–2014. The basic characteristics of NHANES 2013–2014 was similar to NHANES 2009–2014 (Appendix Table 8). We explored the association of PFOS and sex hormones, and sex hormones with periodontitis, respectively, and found PFOS positively associated with testosterone and TT/E_2_ (*P* < 0.05) (Appendix Table 9). The results of logistic regression of sex hormones with periodontitis showed that TT/E_2_ was associated with a higher risk of periodontitis (*P* < 0.05) (Appendix Table 10). The mediation analysis revealed that 37.4% and 16.5% of this association was mediated by testosterone in middle and high levels of PFOS respectively (compared to low tertiles). Moreover, TT/E_2_ was found to mediate 61.2% and 31.7% of the association in middle and high levels of PFOS. Notably, the indirect effect was statistically significant, as indicated by the 95% CI not encompassing zero. However, no significant mediation effects by estradiol and SHBG were observed (Fig. 3). Additionally, we conducted an examination of the reverse associations (i.e., PFOS as the mediator of sex hormone’s effect on periodontitis). Our findings indicated that the reverse mediation effect was not statistically significant, providing additional support for the robustness of the mediation analysis results.

### Subgroup and sensitivity analyses

Subgroup analysis showed that there was basically no interaction between seven PFASs and covariates, and all *P*-values for interaction were virtually greater than 0.05 (Appendix Table 11, and 12).

To further confirm the robustness of the results, we conducted a number of sensitivity analyses by using periodontal clinical indices (mean AL, mean PPD, and the number of teeth) as dependent variables. As shown in Appendix Table 13, in the fully adjusted model, significant positive associations were observed between high levels of PFOS, PFUA and mean AL. Also, significant positive associations were found between high levels of PFOS, PFNA and mean PPD (*P* < 0.05). Conversely, negative associations were found between high levels of PFOS, PFNA and the number of teeth (*P* < 0.05). Furthermore, our findings indicated significant mediation effects of sex hormones on the relationship between PFOS levels and mean AL or mean PPD. Specifically, testosterone explained 55.1% and 44.4% of the association between middle and high levels of PFOS and AL, and TT/E_2_ were found to mediate 91.3% and 80.4% of these associations (Appendix Fig. 5). Similarly, testosterone and TT/E_2_ mediated 42.8% and 45.6% of the association between a high level of PFOS and PPD, respectively (Appendix Fig. 6). We also conducted a sensitivity analysis by removing those with missing values, the strengths and directions of point estimates were largely unchanged, although the 95% confidence intervals for certain PFASs became a slightly wider (Appendix Table 14). Additionally, the results, presented in Appendix Table 15, indicated E-values of 1.41 for high levels of PFOS and 1.38 for PFNA. Given the adjustment for various potential confounders in this study, it is almost impossible or difficult to exist such strong unmeasured confounders. Therefore, the observed association between PFOS or PFNA and periodontitis cannot be completely negated by unmeasured confounders.

## Discussion

To our knowledge, this is the first study to report an association between serum PFAS levels and periodontitis. Based on a large sample of participants from the NHANES 2009–2014, high serum concentrations of PFOS and PFNA were significantly associated with periodontitis. The BKMR model also confirmed a positive association between PFAS mixtures and periodontitis. Of note, sex hormones may partially mediate the relationship between PFOS levels and periodontitis.

Data on the epidemiology of PFAS levels in periodontitis are scarce. PFASs are a new class of POPs, prior studies on the relationship between POPs and periodontitis may indirectly support the findings of this study. Exposure to POPs was positively related to periodontitis, which may increase susceptibility to bacterial infection in the periodontium due to immunological dysfunction [33]. Previous animal research on mink showed that exposure to POPs could induce the proliferation of squamous epithelium in the periodontal ligament, alveolar bone resorption, and subsequent loose teeth [34].

The association of PFAS with sex hormones found in our study, and the association of sex hormones with periodontitis are in general agreement with previous studies [14, 35]. The biological mechanisms by which PFASs may affect periodontitis have not yet been studied. In our study, we observed significant mediation effects of testosterone and TT/E_2_ on the relationship between specific PFASs and periodontitis. PFASs are a class of endocrine-disrupting chemicals [36], and exposure to PFASs was found to increase androgen levels and reduce estrogen levels [37, 38]. Although normal androgen levels play an important role in the balance of the skeletal internal environment and can inhibit bone resorption [39], it has been reported that excessive androgens have a detrimental effect on bone synthesis and metabolism in women [40]. Given that alveolar bone loss is a sign of periodontitis and is regulated by various hormones and osteocyte mediators [41], it is reasonable to speculate that PFASs may interfere with bone metabolism by altering sex hormone metabolism, ultimately promoting periodontitis.

In addition to disrupting sex hormone metabolism, the association of PFAS levels with periodontitis may also be explained by the marked influence of these compounds on the function of many of the cellular, subcellular, or molecular components of the immune system [42]. Periodontitis is a chronic multifactorial inflammatory disease involving innate and adaptive immune responses and a spectrum of inflammatory cytokines [43]. PFOS and PFOA were reported to exhibit statistically significant in vitro effects on monocyte differentiation, NK-cell function, and cytokine release [44], providing favorable conditions for persistent infection and the development of periodontitis [45, 46]. Because of the unavailability of information on immune cells in the NHANES, we were unable to explore these mediation effects. Further studies are needed to confirm these possibilities.

Our study has some major advantages. First, we weighted our data analysis according to the official guidelines, which allows us to generalize our findings to the general US population. Second, our study included a relatively large sample size of 3248 participants, which ensured the reliability of the results. However, our study also has certain limitations. First, our study population was obtained from the NHANES 2009–2014 database; these populations may not have the same levels of exposure to perfluorinated compounds as today, so the results of this study may not be fully representative of the current exposure levels. Second, periodontal data from the third molars were not included in the analyses because the third molars were not probed in the 2009–2014 cycles of the NHANES. Third, although we adjusted the model using several possible confounding variables, we cannot exclude residual confounding effects from other confounding factors, such as PFAS contamination of soil and groundwater in the residential locations of participants, occupational exposure, and variation in host metabolic capabilities. Fourthly, we used tertiles of PFAS concentrations instead of a threshold since there is no standardized safety limit of serum PFAS levels. Finally, in our mediation analyses, we specifically utilized the NHANES 2013–2014 survey as it provided complete sex hormone data, potentially impacting the observed lack of statistical significance in the total effect. Furthermore, the NHANES 2013–2014 survey exhibited a substantial prevalence of missing data in relation to the determination of menopause status (50.1%) and hormone therapy (50.3%), thereby precluding their inclusion in the analysis.

## Conclusion

This large population-based study provides preliminary evidence that certain PFASs (PFOS and PFNA) are associated with periodontitis, and this association may be partially mediated by sex hormones. Our findings provide a new perspective on the pathogenesis of periodontitis and serve as a call for action to increase attention to PFAS exposure in periodontitis. More prospective studies and mechanism-based studies are needed for further exploration of this relationship in the future.

### Electronic supplementary material

Below is the link to the electronic supplementary material.

Supplementary Material 1

## Data Availability

National Health and Nutrition Examination Survey (NHANES) data supporting the conclusions of this research are accessible at https://www.cdc.gov/nchs/nhanes/index.htm.
