# Supplementary material for: Association of per- and polyfluoroalkyl substances (PFAS) with periodontitis: the mediating role of sex hormones
Source: BMC Oral Health. 2024 Feb 15;24:243. doi: 10.1186/s12903-024-03863-0 (PMC10870532; doi:10.1186/s12903-024-03863-0)
Supplement: Supplementary file 1 — Supplementary Material 1 [file 12903_2024_3863_MOESM1_ESM.docx]

**Supplementary materials**

**Appendix Table 1** Detection frequencies of serum PFAS in NHANES 2009-2014 (N = 3248).

**Appendix Table 2** Code for weight adjustment procedure in NHANES 2009-2014 (N = 3248).

**Appendix Table 3** Variance inflation factor (VIF) of covariates in multivariable logistic regression models in NHANES 2009-2014 (N= 3248).

**Appendix Table 4** PFASs of total population in NHANES 2009-2014 (N= 3248).

**Appendix Table 5** PFASs of participants with different periodontal conditions in NHANES 2009-2014 (N= 3248).

**Appendix Table 6** The characteristics of participants with different periodontal conditions in NHANES 2009-2014 (N = 3248).

**Appendix Table 7** Results of unordered multivariable logistic regression analysis in NHANES 2009-2014 (N = 3248).

**Appendix Table 8** Demographic characteristics of the population in NHANES 2013-2014 (N = 1055).

**Appendix Table 9** Association of seven PFOS with sex hormones (β (95%CI)) in NHANES 2013–2014 (N = 1055).

**Appendix Table 10** Association between sex hormones and periodontitis in NHANES 2013-2014 (n = 1055).

**Appendix Table 11** Interaction between PFOS and general characteristics in NHANES 2009-2014 (N = 3248).

**Appendix Table 12** Interaction between PFNA and general characteristics in NHANES 2009-2014 (N = 3248).

**Appendix Table 13** Association of seven PFASs with periodontal clinical indices in NHANES 2009–2014 (N = 3248).

**Appendix Table 14** PRs (95%CIs) for PFASs and periodontitis risk in the NHANES from 2009–2014 (Data without missing values in covariates) (N = 2736).

**Appendix Table 15** E-values and lower limit of 95% CIs for the association between PFAS and periodontitis.

**Appendix Figure 1** Directed acyclic graph of confounding factors.

**Appendix Figure 2** Schematic of a simple mediation model.

**Appendix Figure 3** Distribution of seven PFASs in NHANES participants (2009-2014).

**Appendix Figure 4** Restricted cubic spline curves of serum PFOS/PFNA concentrations in NHANES 2009-2014 (N = 3248).

**Appendix Figure 5** Mediation analyses of association between PFOS and attachment loss (AL).

**Appendix Figure 6** Mediation analyses of association between PFOS and periodontal probing depth (PPD).

**Appendix Table 1** Detection frequencies of serum PFAS in NHANES 2009-2014 (N = 3248).

| Survey cycle | 2009-2010 n = 1200 | | 2011-2012 n = 993 | | 2013-2014 n = 1055 | | Overall |  |
| --- | --- | --- | --- | --- | --- | --- | --- | --- |
|  | LLOD | n (%)  >LLOD | LLOD | n (%)  >LLOD | LLOD | n (%)  >LLOD | n (%)  >LLOD |  |
|  |  |  |  |  |  |  |  |  |
| PFOA | 0.10 | 1196(99.7) | 0.10 | 989(99.6) | - | - | 3239(99.7) |  |
| PFOS | 0.20 | 1197(99.8) | 0.20 | 989(99.6) | - | - | 3232(99.5) |  |
| PFHxS | 0.10 | 1191(99.3) | 0.10 | 979(98.6) | 0.10 | 1042(98.8) | 3212(98.9) |  |
| MPAH | 0.20 | 900(75.0) | 0.09 | 535(53.9) | 0.10 | 482(45.7) | 1917(59.0) |  |
| PFDE | 0.10 | 1143(95.3) | 0.10 | 870(87.6) | 0.10 | 875(82.9) | 2888(88.9) |  |
| PFNA | 0.08 | 1198(99.8) | 0.08 | 986(99.3) | 0.10 | 1043(98.9) | 3227(99.4) |  |
| PFUA | 0.10 | 908(75.7) | 0.10 | 677(68.2) | 0.10 | 545(51.7) | 2130(65.6) |  |
| n-PFOA |  |  |  |  | 0.10 | 1048(99.3) |  |  |
| Sb-PFOA |  |  |  |  | 0.10 | 879(83.3) |  |  |
| n-PFOS |  |  |  |  | 0.10 | 1047(99.2) |  |  |
| Sm-PFOS |  |  |  |  | 0.10 | 1037(98.3) |  |  |

Note: LLOD: The lower limit of detection (in ng/mL).

N-PFOS refers to the linear perfluorooctane sulfonic acid isomers, while Sm-PFOS means branched perfluorooctane sulfonic acid isomers. PFOS encompasses the combined total of n-PFOS and Sm-PFOS.

**Appendix Table 2** Code for weight adjustment procedure in NHANES 2009-2014 (N = 3248).

| Combining 3 cycles (6 years) | |
| --- | --- |
| Cycle of NHANES | Combined weights |
| 2009-2010 | 1/3 * WTSC2YR |
| 2011-2012 | 1/3 * WTSA2YR |
| 2013-2014 | 1/3 * WTSB2YR |
|  | 1/3 * WTSBBN2Y |

Note: Four perfluoroalkyl and polyfluoroalkyl substances (PFAS) isomers were analyzed using surplus serum specimens from NHANES 2013-2014: n-PFOA, Sb-PFOA, n-PFOS, Sm-PFOS, which makes the weights different.

**Appendix Table 3** Variance inflation factor (VIF) of covariates in multivariable logistic regression models in NHANES 2009-2014 (N= 3248).

| Variables | PFOA | PFOS | PFNA | PFHxS | MPAH | PFDE | PFUA |
| --- | --- | --- | --- | --- | --- | --- | --- |
| PFAS | 1.206 | 1.165 | 1.147 | 1.213 | 1.153 | 1.167 | 1.177 |
| Age | 1.267 | 1.248 | 1.193 | 1.201 | 1.189 | 1.196 | 1.208 |
| Sex | 1.174 | 1.228 | 1.148 | 1.273 | 1.113 | 1.121 | 1.154 |
| BMI (kg/m^2^) | 1.208 | 1.193 | 1.225 | 1.234 | 1.193 | 1.217 | 1.282 |
| Race/ethnicity | 1.103 | 1.120 | 1.124 | 1.123 | 1.198 | 1.136 | 1.125 |
| Educational attainment | 1.242 | 1.277 | 1.253 | 1.383 | 1.287 | 1.242 | 1.269 |
| Marital status | 1.215 | 1.189 | 1.205 | 1.186 | 1.182 | 1.202 | 1.172 |
| Smoking status | 1.662 | 1.783 | 1.713 | 1.687 | 1.657 | 1.753 | 1.678 |
| Alcohol intake (drinks/day) | 1.260 | 1.235 | 1.282 | 1.220 | 1.185 | 1.285 | 1.231 |
| Poverty income ratio | 1.160 | 1.179 | 1.191 | 1.197 | 1.151 | 1.168 | 1.206 |
| Serum cotinine (ng/mL) | 1.521 | 1.618 | 1.538 | 1.597 | 1.524 | 1.527 | 1.594 |
| Milk product consumption | 1.256 | 1.188 | 1.250 | 1.194 | 1.172 | 1.229 | 1.226 |

**Appendix Table 4** PFASs of total population in NHANES 2009-2014 (N= 3248).

| Variables  (ng/mL) | Total  (Medians and quartiles) N = 3248 | Participants with no periodontitis  (Medians and quartiles) n=1605 (58.8%) | Participants with periodontitis  (Medians and quartiles) n=1643 (41.2%) | *P* value |
| --- | --- | --- | --- | --- |
| PFOA | 2.47(1.60,3.70) | 2.47(1.60,3.67) | 2.50(1.60,3.79) | 0.880 |
| PFOS | 7.64(4.46,12.30) | 7.16(4.14,11.70) | 8.30(4.80,13.10) | 0.004 |
| PFHXS | 1.50(0.80,2.50) | 1.40(0.80,2.50) | 1.50(0.90,2.50) | 0.362 |
| MPAH | 0.10(0.07,0.30) | 0.10(0.07,0.30) | 0.11(0.07,0.30) | 0.216 |
| PFUA | 0.10(0.07,0.24) | 0.10(0.07,0.23) | 0.11(0.07,0.26) | 0.566 |
| PFNA | 0.94(0.62,1.48) | 0.90(0.60,1.40) | 0.98(0.66,1.56) | 0.036 |
| PFDE | 0.20(0.13,0.39) | 0.20(0.14,0.39) | 0.20(0.13,0.38) | 0.812 |
| PFOA [tertile (ng/mL)] |  |  |  | 0.852 |
| Low (＜1.87) | 1118(33.3) | 558(33.0) | 560(33.6) |  |
| Middle (1.87-3.17) | 1048(31.8) | 515(31.6) | 533(32.1) |  |
| High (≥3.17) | 1082(35.0) | 532(35.4) | 550(34.3) |  |
| PFOS [tertile (ng/mL)] |  |  |  | 0.010 |
| Low (＜5.40） | 1104(33.9) | 604(35.8) | 500(31.0) |  |
| Middle (5.40-10.90) | 1070(35.4) | 552(36.5) | 518(33.8) |  |
| High (≥10.90） | 1074(30.7) | 449(27.6) | 625(35.2) |  |
| PFHxS [tertile (ng/mL)] |  |  |  | 0.202 |
| Low (＜1.00） | 1113(33.2) | 602(34.6) | 511(31.2) |  |
| Middle (1.00-2.07) | 1053(32.5) | 492(31.3) | 561(34.4) |  |
| High (≥2.07） | 1082(34.3) | 511(34.2) | 571(34.5) |  |
| MPAH [tertile (ng/mL)] |  |  |  | 0.074 |
| Low (＜0.07） | 1331(40.0) | 672(41.0) | 659(38.7) |  |
| Middle (0.07-0.20) | 942(29.9) | 494(31.0) | 448(28.3) |  |
| High (≥0.20） | 975(30.1) | 439(28.0) | 536(33.0) |  |
| PFUA [tertile (ng/mL)] |  |  |  | 0.426 |
| Low (＜0.07） | 1118(37.6) | 561(37.7) | 557(37.4) |  |
| Middle (0.07-0.20) | 1135(35.0) | 582(36.1) | 553(33.5) |  |
| High (≥0.20） | 995(27.4) | 462(26.2) | 533(29.1) |  |
| PFNA [tertile (ng/mL)] |  |  |  | 0.036 |
| Low (＜0.74） | 1085(35.2) | 571(37.2) | 514(32.3) |  |
| Middle (0.74-1.30) | 1091(33.9) | 563(34.0) | 528(33.7) |  |
| High (≥1.30） | 1072(30.9) | 471(28.8) | 601(34.0) |  |
| PFDE [tertile (ng/mL)] |  |  |  | 0.796 |
| Low (＜0.20) | 1610(51.6) | 822(52.2) | 788(50.6) |  |
| Middle (0.20-0.30) | 626(20.0) | 307(19.7) | 319(20.3) |  |
| High (≥0.30） | 1012(28.5) | 476(28.1) | 536(29.0) |  |

Note: Medians and quartiles were weight-adjusted using NHANES-specified sampling weights.

We used Mann-Whitney U test or chi-squared test to compare PFASs levels between two groups.

**Appendix Table 5** PFASs of participants with different periodontal conditions in NHANES 2009-2014 (N= 3248).

| Variables  (ng/mL) | Total N=3248 | No periodontitis N=1605 (49.4%) | Mild periodontitis N=154 (4.7%) | Moderate periodontitis N=1157 (35.6%) | Severe periodontitis N=332 (10.2%) | *P* value |
| --- | --- | --- | --- | --- | --- | --- |
| PFOA | 2.47(1.60,3.70) | 2.47(1.60,3.67) | 2.70(2.00,3.80) | 2.40(1.56,3.70) | 2.40(1.60,3.90) | 0.230 |
| PFOS | 7.64(4.46,12.30) | 7.16(4.14,11.70) | 9.50(6.40,12.50) | 8.20(4.70,13.10) | 8.20(5.00,13.40) | **0.010** |
| PFHXS | 1.50(0.80,2.50) | 1.40(0.80,2.50) | 1.50(0.90,2.41) | 1.50(0.90,2.50) | 1.70(1.00,2.70) | 0.316 |
| MPAH | 0.10(0.07,0.30) | 0.10(0.07,0.30) | 0.10(0.06,0.30) | 0.12(0.07,0.30) | 0.10(0.06,0.30) | 0.610 |
| PFUA | 0.10(0.07,0.24) | 0.10(0.07,0.23) | 0.14(0.07,0.28) | 0.11(0.07,0.24) | 0.10(0.07,0.30) | 0.891 |
| PFNA | 0.94(0.62,1.48) | 0.90(0.60,1.40) | 1.05(0.74,1.56) | 0.98(0.61,1.55) | 1.00(0.66,1.56) | 0.093 |
| PFDE | 0.20(0.13,0.39) | 0.20(0.14,0.39) | 0.22(0.20,0.40) | 0.20(0.12,0.37) | 0.20(0.12,0.40) | 0.678 |
| PFOA [tertile (ng/mL)] |  |  |  |  |  | 0.147 |
| Low (＜1.87) | 1118(33.3) | 558(33.0) | 40(21.8) | 413(36.2) | 107(30.2) |  |
| Middle (1.87-3.17) | 1048(31.8) | 515(31.6) | 55(39.5) | 368(30.4) | 110(34.5) |  |
| High (≥3.17) | 1082(35.0) | 532(35.4) | 59(38.7) | 376(33.4) | 115(35.3) |  |
| PFOS [tertile (ng/mL)] |  |  |  |  |  | **0.007** |
| Low (＜5.40） | 1104(33.9) | 604(35.8) | 48(23.2) | 358(32.4) | 94(30.5) |  |
| Middle (5.40-10.90) | 1070(35.4) | 552(36.5) | 46(34.5) | 369(33.3) | 103(35.2) |  |
| High (≥10.90） | 1074(30.7) | 449(27.6) | 60(42.3) | 430(34.3) | 135(34.3) |  |
| PFHxS [tertile (ng/mL)] |  |  |  |  |  | 0.358 |
| Low (＜1.00） | 1113(33.2) | 602(34.6) | 61(32.8) | 362(32.2) | 88(25.5) |  |
| Middle (1.00-2.07) | 1053(32.5) | 492(31.3) | 44(31.4) | 392(34.1) | 125(37.7) |  |
| High (≥2.07） | 1082(34.3) | 511(34.2) | 49(35.8) | 403(33.8) | 119(36.8) |  |
| MPAH [tertile (ng/mL)] |  |  |  |  |  | 0.269 |
| Low (＜0.07） | 1331(40.0) | 672(41.0) | 56(34.8) | 469(39.6) | 134(37.1) |  |
| Middle (0.07-0.20) | 942(29.9) | 494(31.0) | 48(29.2) | 312(28.1) | 88(28.6) |  |
| High (≥0.20） | 975(30.1) | 439(28.0) | 50(36.1) | 376(32.3) | 110(34.3) |  |
| PFUA [tertile (ng/mL)] |  |  |  |  |  | 0.486 |
| Low (＜0.07） | 1118(37.6) | 561(37.7) | 48(33.0) | 392(37.6) | 117(39.5) |  |
| Middle (0.07-0.20) | 1135(35.0) | 582(36.1) | 63(38.9) | 391(33.9) | 99(27.8) |  |
| High (≥0.20） | 995(27.4) | 462(26.2) | 43(28.0) | 374(28.5) | 116(32.7) |  |
| PFNA [tertile (ng/mL)] |  |  |  |  |  | 0.089 |
| Low (＜0.74） | 1085(35.2) | 571(37.2) | 43(26.1) | 363(33.7) | 108(30.4) |  |
| Middle (0.74-1.30) | 1091(33.9) | 563(34.0) | 52(37.4) | 381(32.9) | 95(34.6) |  |
| High (≥1.30） | 1072(30.9) | 471(28.8) | 59(36.4) | 413(33.4) | 129(35.0) |  |
| PFDE [tertile (ng/mL)] |  |  |  |  |  | 0.558 |
| Low (＜0.20) | 1610(51.6) | 822(52.2) | 72(45.6) | 553(51.0) | 163(52.4) |  |
| Middle (0.20-0.30) | 626(20.0) | 307(19.7) | 39(26.6) | 227(20.4) | 53(15.9) |  |
| High (≥0.30） | 1012(28.5) | 476(28.1) | 43(27.8) | 377(28.6) | 116(31.7) |  |

Notes: We compared PFASs levels among different periodontal conditions using Kruskal-Wallis H test or chi-squared test.

**Appendix Table 6** The characteristics of participants with different periodontal conditions in NHANES 2009-2014 (N = 3248).

| Variable | Total N=3248 | No periodontitis N=1605 (49.4%) | Mild periodontitis N=154 (4.7%) | Moderate periodontitis N=1157 (35.6%) | Severe periodontitis N=332 (10.2%) | *P* value |
| --- | --- | --- | --- | --- | --- | --- |
| Continuous variables, mean (SD) |  |  |  |  |  |  |
| Age (years) | 50.79(0.31) | 48.21(0.49) | 48.31(1.18) | 55.47(0.51) | 54.36(0.93) | < 0.001 |
| Alcohol intake (drinks/day) | 2.47(0.06) | 2.24(0.06) | 2.68(0.28) | 2.78(0.13) | 2.96(0.20) | < 0.001 |
| Poverty income ratio, PIR | 3.14(0.05) | 3.45(0.06) | 3.05(0.18) | 2.76(0.06) | 2.26(0.14) | < 0.001 |
| Serum cotinine (ng/mL) | 49.42(3.11) | 33.30(2.97) | 29.92(8.21) | 67.60(5.43) | 122.39(13.53) | < 0.001 |
| Categorical variables, n (%) |  |  |  |  |  |  |
| Age group |  |  |  |  |  | < 0.001 |
| <65 years old | 2538(82.1) | 1359(86.6) | 138(88.5) | 789(72.6) | 252(80.9) |  |
| ≥ 65 years old | 710(17.9) | 246(13.4) | 16(11.5) | 368(27.4) | 80(19.1) |  |
| Sex |  |  |  |  |  | < 0.001 |
| Male | 1599(49.2) | 657(43.3) | 80(53.9) | 630(55.4) | 232(69.4) |  |
| Female | 1649(50.8) | 948(56.7) | 74(46.1) | 527(44.6) | 100(30.6) |  |
| BMI (kg/m^2^) |  |  |  |  |  | 0.503 |
| Normal (< 25.0) | 881(27.5) | 454(28.8) | 29(20.9) | 312(26.9) | 86(22.9) |  |
| Overweight (25.0-29.9) | 1139(35.3) | 559(35.2) | 57(38.5) | 404(34.4) | 119(37.8) |  |
| Obese (≥ 30.0) | 1228(37.3) | 592(36.0) | 68(40.6) | 441(38.8) | 127(39.3) |  |
| Race/ethnicity |  |  |  |  |  | < 0.001 |
| Non-Hispanic White | 1418(69.1) | 832(75.8) | 53(58.0) | 442(62.2) | 91(49.0) |  |
| Non-Hispanic Black | 627(9.9) | 237(7.2) | 34(13.8) | 246(11.8) | 110(23.0) |  |
| Mexican American | 471(8.1) | 156(5.0) | 34(12.4) | 211(12.1) | 70(14.8) |  |
| Other | 732(12.8) | 380(12.0) | 33(15.9) | 258(14.0) | 61(13.3) |  |
| Educational attainment |  |  |  |  |  | < 0.001 |
| Less than High school | 792(15.9) | 229( 9.2) | 45(18.2) | 389(25.1) | 129(32.2) |  |
| High school and college or above | 2456(84.1) | 1376(90.8) | 109(81.8) | 768(74.9) | 203(67.8) |  |
| Marital status |  |  |  |  |  | < 0.001 |
| Married | 1945(64.2) | 1035(69.4) | 88(62.2) | 647(57.3) | 175(51.5) |  |
| Unmarried but have/had partner | 937(25.6) | 382(20.9) | 46(24.8) | 395(32.6) | 114(35.9) |  |
| Never married | 366(10.2) | 188(9.7) | 20(13.0) | 115(10.1) | 43(12.5) |  |
| Smoking status |  |  |  |  |  | < 0.001 |
| Never | 1858(57.3) | 1035(63.7) | 107(69.4) | 582(47.7) | 134(35.4) |  |
| Former | 813(25.7) | 368(24.6) | 24(16.5) | 333(29.6) | 88(24.5) |  |
| Current | 577(17.0) | 202(11.7) | 23(14.1) | 242(22.6) | 110(40.2) |  |
| Milk product consumption |  |  |  |  |  | 0.585 |
| Milk product consumption | 585(16.8) | 279(16.4) | 28(17.8) | 209(17.1) | 69(17.8) |  |
| Rarely-less than once a week | 508(14.9) | 271(15.8) | 28(15.0) | 155(13.3) | 54(14.8) |  |
| Sometimes-once a week or more, but less than once a day | 910(27.2) | 456(27.7) | 50(32.1) | 314(25.0) | 90(28.7) |  |
| Often-once a day or more | 1231(40.6) | 592(39.5) | 48(35.0) | 474(44.4) | 117(38.3) |  |
| Varied | 14( 0.4) | 7(0.5) | 0(0.0) | 5(0.3) | 2(0.4) |  |

Notes: We compared demographic characteristics between the periodontitis and non-periodontitis groups using the chi-squared test or Kruskal-Wallis H test.

**Appendix Table 7** Results of unordered multivariable logistic regression analysis in NHANES 2009-2014 (N = 3248).

| PFASs | No periodontitis N=1605 (49.4%) | Mild periodontitis N=154 (4.7%) | | Moderate periodontitis N=1157 (35.6%) | | Severe periodontitis N=332 (10.2%) | |
| --- | --- | --- | --- | --- | --- | --- | --- |
|  |  | OR (95%CI) | *P* value | OR (95%CI) | *P* value | OR (95%CI) | *P* value |
| PFOA [tertile (ng/mL)] |  |  |  |  |  |  |  |
| Low (＜1.87) | Ref | Ref | Ref | Ref | Ref | Ref | Ref |
| Middle (1.87-3.17) | Ref | 1.61 (1.04, 2.50) | 0.034 | 0.87 (0.71, 1.07) | 0.187 | 0.97 (0.71, 1.34) | 0.874 |
| High (≥3.17) | Ref | 1.86 (1.18, 2.92) | **0.007** | 0.84 (0.69, 1.04) | 0.115 | 1.02 (0.73, 1.40) | 0.924 |
| PFOS [tertile (ng/mL)] |  |  |  |  |  |  |  |
| Low (＜5.40） | Ref | Ref | Ref | Ref | Ref | Ref | Ref |
| Middle (5.40-10.90) | Ref | 1.19 (0.77, 1.84) | 0.435 | 1.09 (0.89, 1.33) | 0.428 | 1.10 (0.79, 1.53) | 0.577 |
| High (≥10.90） | Ref | 1.95 (1.26, 3.01) | **0.003** | 1.46 (1.18, 1.81) | **<0.001** | 1.74 (1.25, 2.42) | **0.001** |
| PFHxS [tertile (ng/mL)] |  |  |  |  |  |  |  |
| Low (＜1.00） | Ref | Ref | Ref | Ref | Ref | Ref | Ref |
| Middle (1.00-2.07) | Ref | 0.84 (0.55, 1.30) | 0.444 | 1.09 (0.88, 1.34) | 0.440 | 1.26 (0.90, 1.75) | 0.174 |
| High (≥2.07） | Ref | 0.95 (0.61, 1.50) | 0.833 | 0.99 (0.79, 1.23) | 0.895 | 1.05 (0.74, 1.49) | 0.775 |
| MPAH [tertile (ng/mL)] |  |  |  |  |  |  |  |
| Low (＜0.07） | Ref | Ref | Ref | Ref | Ref | Ref | Ref |
| Middle (0.07-0.20) | Ref | 1.24 (0.83, 1.88) | 0.296 | 0.83 (0.68, 1.02) | 0.076 | 0.79 (0.58, 1.09) | 0.148 |
| High (≥0.20） | Ref | 1.59 (1.05, 2.41) | **0.027** | 1.09 (0.89, 1.33) | 0.397 | 1.11 (0.82, 1.51) | 0.493 |
| PFUA [tertile (ng/mL)] |  |  |  |  |  |  |  |
| Low (＜0.07） | Ref | Ref | Ref | Ref | Ref | Ref | Ref |
| Middle (0.07-0.20) | Ref | 1.38 (0.92, 2.06) | 0.118 | 1.03 (0.84, 1.25) | 0.794 | 0.89 (0.65, 1.22) | 0.477 |
| High (≥0.20） | Ref | 1.28 (0.82, 2.01) | 0.280 | 1.32 (1.07, 1.63) | **0.009** | 1.49 (1.09, 2.04) | 0.013 |
| PFNA [tertile (ng/mL)] |  |  |  |  |  |  |  |
| Low (＜0.74） | Ref | Ref | Ref | Ref | Ref | Ref | Ref |
| Middle (0.74-1.30) | Ref | 1.27 (0.83, 1.95) | 0.275 | 1.03 (0.84, 1.26) | 0.786 | 0.84 (0.61, 1.15) | 0.278 |
| High (≥1.30） | Ref | 1.83 (1.20, 2.80) | **0.005** | 1.34 (1.10, 1.65) | **0.005** | 1.43 (1.05, 1.95) | **0.022** |
| PFDE [tertile (ng/mL)] |  |  |  |  |  |  |  |
| Low (＜0.20) | Ref | Ref | Ref | Ref | Ref | Ref | Ref |
| Middle (0.20-0.30) | Ref | 1.58 (1.04, 2.41) | **0.032** | 1.20 (0.96, 1.49) | 0.105 | 0.95 (0.66, 1.35) | 0.761 |
| High (≥0.30） | Ref | 1.15 (0.77, 1.73) | 0.500 | 1.25 (1.03, 1.51) | **0.024** | 1.38 (1.03, 1.83) | **0.030** |

Notes: Ref = Reference. Adjusted by age, sex, race/ethnicity, educational attainment, marital status, BMI, smoking status, alcohol intake, milk product consumption, poverty income ratio, and serum cotinine.

**Appendix Table 8** Basic characteristics of the population in NHANES 2013-2014 (N = 1055).

| Characteristics | Total N=1055 | Participants with no periodontitis N=585 (55.5%) | Participants with periodontitis N=470 (44.5%) | *P* value |
| --- | --- | --- | --- | --- |
| Continuous variables, mean (SD) |  |  |  |  |
| Age (years) | 51.62(0.64) | 50.15(1.00) | 54.32(0.72) | 0.008 |
| Alcohol intake (drinks/day) | 2.38(0.11) | 2.16(0.11) | 2.78(0.14) | < 0.001 |
| Poverty income ratio, PIR | 3.10(0.13) | 3.41(0.14) | 2.53(0.11) | < 0.001 |
| Serum cotinine (ng/mL) | 54.17(7.25) | 43.59(7.33) | 73.47(7.49) | < 0.001 |
| Categorical variables, n (%) |  |  |  |  |
| Age group |  |  |  | 0.010 |
| Youth and middle (Age ˂ 65) | 809(79.8) | 479(81.7) | 330(76.3) |  |
| Old (Age ≥ 65) | 246(20.2) | 106(18.3) | 140(23.7) |  |
| Sex |  |  |  | < 0.001 |
| Female | 556(50.9) | 340(54.3) | 216(44.5) |  |
| Male | 499(49.1) | 245(45.7) | 254(55.5) |  |
| BMI (kg/m^2^) |  |  |  |  |
| Normal | 279(24.1) | 157(24.8) | 122(22.9) |  |
| Overweight | 383(37.3) | 217(38.5) | 166(35.1) | < 0.001 |
| Obese | 393(38.6) | 211(36.7) | 182(42.0) |  |
| Race/ethnicity |  |  |  |  |
| Mexican American | 149( 9.1) | 51( 5.3) | 98(16.0) |  |
| Non-Hispanic Black | 183( 9.1) | 74( 6.4) | 109(14.1) | 0.001 |
| Non-Hispanic White | 461(68.3) | 295(74.9) | 166(56.2) |  |
| Other | 262(13.5) | 165(13.4) | 97(13.7) |  |
| Educational attainment |  |  |  |  |
| Less than High school | 168(22.28) | 51(8.44) | 117(28.74) | 0.177 |
| High school and college or above | 586(77.72) | 360(91.56) | 226(71.26) |  |
| Marital status |  |  |  |  |
| Married | 645(66.5) | 395(72.9) | 250(54.7) | 0.517 |
| Never married | 109( 8.5) | 57( 7.5) | 52(10.3) |  |
| Unmarried but have/had partner | 301(25.1) | 133(19.6) | 168(35.0) |  |
| Smoking status |  |  |  |  |
| Former | 257(25.5) | 131(23.9) | 126(28.5) | < 0.001 |
| Never | 606(56.6) | 370(61.9) | 236(47.0) |  |
| Current | 192(17.8) | 84(14.1) | 108(24.5) |  |
| Milk product consumption |  |  |  | 0.424 |
| Never | 201(17.8) | 124(19.9) | 77(13.9) |  |
| Rarely-less than once a week | 177(16.0) | 105(16.5) | 72(15.1) |  |
| Sometimes-once a week or more, but less than once a day | 295(25.9) | 159(25.2) | 136(27.1) |  |
| Often-once a day or more | 377(39.8) | 195(37.9) | 182(43.3) |  |
| Varied | 5( 0.5) | 2(0.5) | 3(0.5) |  |

Notes: We compared demographic characteristics between the periodontitis and non-periodontitis groups using the chi-squared test or Mann‒Whitney U test.

**Appendix Table 9** Association of PFOS with sex hormones (β (95%CI)) in NHANES 2013–2014 (N = 1055).

| PFAS | Testosterone | | Estradiol | | SHBG | | TT/E2 | |
| --- | --- | --- | --- | --- | --- | --- | --- | --- |
|  | β(95%CI) | *P* value | β(95%CI) | *P* value | β(95%CI) | *P* value | β(95%CI) | *P* value |
| PFOS [tertile (ng/mL)] |  |  |  |  |  |  |  |  |
| Low (＜5.40） | ref | ref | ref | ref | ref | ref | ref | ref |
| Middle (5.40-10.90) | 0.95( 0.58,1.31) | **0.004** | -0.1(-0.44, 0.23) | 0.402 | -0.1(-0.30, 0.10) | 0.208 | 1.06( 0.48,1.63) | **0.010** |
| High (≥10.90） | 1.15( 0.65,1.64) | **0.005** | -0.1(-0.40, 0.20) | 0.354 | -0.07(-0.28, 0.14) | 0.381 | 1.29( 0.78,1.80) | **0.004** |

**Notes:** Adjusted by age, race/ethnicity, educational attainment, marital status, BMI, smoking status, alcohol intake, milk product consumption, poverty income ratio, and serum cotinine. Abbreviation: SHBG: sex hormone binding globulin; TT/E_2_: a ratio of testosterone to estradiol; an indirect assessment of circulating free testosterone.

**Appendix Table 10** Association between sex hormones and periodontitis in NHANES 2013-2014 (n = 1055).

| Sex hormones | OR (95%CI) | *P* value |
| --- | --- | --- |
| Testosterone | 1.07(0.99,1.16) | 0.084 |
| Estradiol | 0.89(0.75,1.06) | 0.893 |
| SHBG | 1.01(0.78,1.31) | 0.924 |
| TT/E2 | 1.11(1.01,1.21) | **0.031** |

Notes: Adjusted by age, race/ethnicity, educational attainment, marital status, BMI, smoking status, alcohol intake, milk product consumption, poverty income ratio, and serum cotinine. Abbreviation: SHBG: sex hormone binding globulin; TT/E_2_: a ratio of testosterone to estradiol; an indirect assessment of circulating free testosterone.

**Appendix Table 11** Interaction between PFOS and general characteristics in NHANES 2009-2014 (N = 3248).

| Variables | PR(95%CI) | | | *P* for trend | *P* for interaction |
| --- | --- | --- | --- | --- | --- |
|  | Low (<5.40) | Middle (5.40-10.90) | High (≥10.90) |  |  |
| Age group |  |  |  |  | 0.701 |
| <65 years old | Ref | 1.11(0.80,1.54) | 1.51(1.07,2.12) | 0.021 |  |
| ≥ 65 years old | Ref | 1.27(0.64,2.49) | 1.45(0.81,2.60) | 0.626 |  |
| Sex |  |  |  |  | 0.432 |
| Male | Ref | 0.93(0.58,1.49) | 1.13(0.73,1.76) | 0.416 |  |
| Female | Ref | 1.22(0.81,1.82) | 1.79(1.20,2.67) | 0.241 |  |
| Race/ethnicity |  |  |  |  | 0.113 |
| Non-Hispanic White | Ref | 1.25(0.79,1.98) | 1.44(0.92,2.26) | 0.103 |  |
| Non-Hispanic Black | Ref | 0.69(0.41,1.17) | 0.87(0.51,1.48) | 0.403 |  |
| Mexican American | Ref | 1.22(0.59,2.51) | 1.73(0.81,3.70) | 0.18 |  |
| Other | Ref | 0.88(0.53,1.45) | 2.32(1.30,4.14) | 0.002 |  |
| Marital status |  |  |  |  | 0.538 |
| Married | Ref | 0.99(0.69,1.42) | 1.18(0.80,1.72) | 0.365 |  |
| Unmarried but have/had partner | Ref | 1.17(0.67,2.06) | 1.96(1.17,3.30) | 0.649 |  |
| Never married | Ref | 1.18(0.53,2.64) | 1.77(0.68,4.62) | 0.259 |  |
| Educational attainment |  |  |  |  | 0.744 |
| Less than High school | Ref | 1.01(0.55,1.85) | 1.33(0.76,2.32) | 0.319 |  |
| High School /GED and College | Ref | 1.15(0.84,1.59) | 1.50(1.06,2.14) | 0.024 |  |
| Smoking status |  |  |  |  | 0.802 |
| Never | Ref | 1.07(0.75,1.51) | 1.58(1.06,2.35) | 0.898 |  |
| Former | Ref | 1.24(0.67,2.30) | 1.33(0.80,2.21) | 0.289 |  |
| Current | Ref | 1.03(0.62,1.71) | 1.22(0.60,2.46) | 0.698 |  |
| BMI (kg/m^2^) |  |  |  |  | 0.153 |
| Normal (< 25.0) | Ref | 1.83(1.01,3.33) | 2.25(1.27,3.98) | 0.007 |  |
| Overweight (25.0-29.9) | Ref | 0.81(0.51,1.30) | 1.23(0.75,2.03) | 0.059 |  |
| Obese (≥30.0) | Ref | 1.06(0.67,1.70) | 1.20(0.74,1.96) | 0.007 |  |
| Diabetes |  |  |  |  | 0.777 |
| No | Ref | 1.13(0.82,1.57) | 1.49(1.07,2.07) | 0.019 |  |
| Yes | Ref | 1.11(0.56,2.24) | 1.25(0.63,2.49) | 0.749 |  |

Notes: Adjusted by age, sex, race/ethnicity, educational attainment, marital status, BMI, smoking status, alcohol intake, milk product consumption, poverty income ratio, and serum cotinine.

**Appendix Table 12** Interaction between PFNA and general characteristics in NHANES 2009-2014 (N = 3248).

| Variables | PR(95%CI) | | | *P* for trend | *P* for interaction |
| --- | --- | --- | --- | --- | --- |
|  | Low (<0.74) | Middle (0.74-1.30) | High (≥1.30) |  |  |
| Age group |  |  |  |  | 0.754 |
| <65 years old | Ref | 1.08(0.84,1.39) | 1.43(1.09,1.88) | 0.053 |  |
| ≥ 65 years old | Ref | 1.24(0.71,2.18) | 1.31(0.77,2.23) | 0.516 |  |
| Sex |  |  |  |  | 0.711 |
| Male | Ref | 0.98(0.67,1.43) | 1.22(0.85,1.76) | 0.167 |  |
| Female | Ref | 1.16(0.87,1.55) | 1.54(1.08,2.19) | 0.155 |  |
| Race/ethnicity |  |  |  |  | 0.447 |
| Non-Hispanic White | Ref | 1.04(0.74,1.44) | 1.28(0.88,1.85) | 0.266 |  |
| Non-Hispanic Black | Ref | 0.91(0.58,1.43) | 1.06(0.67,1.67) | 0.496 |  |
| Mexican American | Ref | 0.91(0.57,1.43) | 1.67(0.79,3.55) | 0.109 |  |
| Other | Ref | 1.49(0.95,2.33) | 2.06(1.21,3.50) | 0.155 |  |
| Marital status |  |  |  |  | 0.351 |
| Married | Ref | 0.95(0.71,1.29) | 1.23(0.87,1.73) | 0.106 |  |
| Unmarried but have/had partner | Ref | 1.06(0.73,1.53) | 1.44(1.02,2.02) | 0.221 |  |
| Never married | Ref | 1.69(0.70,4.05) | 1.91(0.87,4.20) | 0.235 |  |
| Educational attainment |  |  |  |  | 0.362 |
| Less than High school | Ref | 0.79(0.41,1.50) | 1.03(0.58,1.84) | 0.973 |  |
| High School /GED and College | Ref | 1.17(0.94,1.45) | 1.46(1.11,1.92) | 0.146 |  |
| Smoking status |  |  |  |  | 0.743 |
| Never | Ref | 1.00(0.76,1.32) | 1.42(1.02,1.97) | 0.995 |  |
| Former | Ref | 1.47(0.95,2.28) | 1.61(1.09,2.38) | 0.709 |  |
| Current | Ref | 0.94(0.44,1.99) | 1.15(0.56,2.39) | 0.728 |  |
| BMI (kg/m^2^) |  |  |  |  | 0.129 |
| Normal (< 25.0) | Ref | 1.39(0.86,2.26) | 1.77(0.98,3.18) | 0.459 |  |
| Overweight (25.0-29.9) | Ref | 1.05(0.71,1.54) | 1.79(1.23,2.60) | 0.003 |  |
| Obese (≥30.0) | Ref | 0.97(0.70,1.35) | 0.94(0.61,1.44) | 0.459 |  |
| Diabetes |  |  |  |  | 0.692 |
| No | Ref | 1.12(0.84,1.48) | 1.38(1.04,1.83) | 0.165 |  |
| Yes | Ref | 0.91(0.52,1.57) | 1.57(0.83,2.97) | 0.071 |  |

Notes: Adjusted by age, sex, race/ethnicity, educational attainment, marital status, BMI, smoking status, alcohol intake, milk product consumption, poverty income ratio, and serum cotinine.

**Appendix Table 13** Association of seven PFASs with periodontal clinical indices in NHANES 2009–2014 (N = 3248).

| PFASs | Attachment loss (AL) | | Periodontal probing depth (PPD) | | Number of teeth | |
| --- | --- | --- | --- | --- | --- | --- |
|  | Unadjusted | Adjusted | Unadjusted | Adjusted | Unadjusted | Adjusted |
| PFOA [tertile (ng/mL)] |  |  |  |  |  |  |
| Low (＜1.87) | Ref | Ref | Ref | Ref | Ref | Ref |
| Middle (1.87-3.17) | 0.03(-0.06,0.11) | -0.02(-0.09, 0.05) | 0.01(-0.05,0.07) | 0.01(-0.05, 0.06) | -0.54(-1.09,0.02) | -0.69(-1.24,-0.14)* |
| High (≥3.17) | 0.02(-0.10,0.14) | -0.01(-0.11, 0.09) | 0.02(-0.06,0.10) | 0.04(-0.04, 0.12) | -0.44(-0.95,0.07) | -0.77(-1.32,-0.21)** |
| PFOS [tertile (ng/mL)] |  |  |  |  |  |  |
| Low (＜5.40） | Ref | Ref | Ref | Ref | Ref | Ref |
| Middle (5.40-10.90) | 0.01(-0.09,0.11) | 0(-0.08, 0.08) | 0.03(-0.02,0.09) | 0.05(-0.01, 0.11) | 0(-0.55, 0.55) | -0.37(-0.83, 0.09) |
| High (≥10.90） | 0.19( 0.07,0.30)** | 0.12( 0.01, 0.24)* | 0.11( 0.03,0.19)* | 0.12( 0.03, 0.21)** | -1.21(-1.76,-0.66)*** | -1.34(-1.94,-0.74)*** |
| PFHxS [tertile (ng/mL)] |  |  |  |  |  |  |
| Low (＜1.00） | Ref | Ref | Ref | Ref | Ref | Ref |
| Middle (1.00-2.07) | 0.15(0.06,0.24)** | 0.02(-0.06, 0.11) | 0.07( 0.01,0.13)* | 0.02(-0.04, 0.08) | -0.61(-1.20,-0.02)* | -0.47(-1.02, 0.07) |
| High (≥2.07） | 0.16(0.04,0.27)** | 0(-0.11, 0.10) | 0.04(-0.03,0.11) | -0.01(-0.08, 0.06) | -0.58(-1.10,-0.05)* | -0.47(-1.05, 0.10) |
| MPAH [tertile (ng/mL)] |  |  |  |  |  |  |
| Low (＜0.07） | Ref | Ref | Ref | Ref | Ref | Ref |
| Middle (0.07-0.20) | -0.01(-0.11,0.08) | -0.05(-0.14, 0.04) | 0.02(-0.04,0.07) | 0.02(-0.03, 0.06) | -0.18(-0.74, 0.39) | 0.06(-0.45, 0.57) |
| High (≥0.20） | 0.14( 0.02,0.26)* | 0.08(-0.02, 0.19) | 0.09( 0.01,0.16)* | 0.09( 0.02, 0.16)* | -0.84(-1.38,-0.30)** | -0.38(-0.86, 0.10) |
| PFUA [tertile (ng/mL)] |  |  |  |  |  |  |
| Low (＜0.07） | Ref | Ref | Ref | Ref | Ref | Ref |
| Middle (0.07-0.20) | -0.09(-0.20,0.02) | -0.04(-0.13, 0.06) | 0(-0.07,0.08) | 0.04(-0.02, 0.11) | 0.08(-0.41,0.57) | -0.27(-0.78, 0.25) |
| High (≥0.20） | 0.06(-0.08,0.19) | 0.15( 0.03, 0.26)* | 0.05(-0.05,0.14) | 0.12( 0.03, 0.21)** | 0.06(-0.46,0.59) | -0.55(-1.15, 0.05) |
| PFNA [tertile (ng/mL)] |  |  |  |  |  |  |
| Low (＜0.74） | Ref | Ref | Ref | Ref | Ref | Ref |
| Middle (0.74-1.30) | 0(-0.09,0.09) | -0.05(-0.13, 0.03) | 0.03(-0.02,0.09) | 0.02(-0.03, 0.08) | -0.42(-0.94,0.09) | -0.41(-0.92, 0.11) |
| High (≥1.30） | 0.11(-0.01,0.23) | 0.09(-0.01, 0.19) | 0.11( 0.01,0.20)* | 0.13( 0.04, 0.21)** | -0.51(-1.06,0.05) | -0.64(-1.17,-0.12)* |
| PFDE [tertile (ng/mL)] |  |  |  |  |  |  |
| Low (＜0.20) | Ref | Ref | Ref | Ref | Ref | Ref |
| Middle (0.20-0.30) | 0.00(-0.11,0.10) | 0.03(-0.07, 0.14) | -0.02(-0.09,0.05) | 0.01(-0.06, 0.07) | -0.1(-0.72,0.52) | -0.43(-1.03, 0.16) |
| High (≥0.30） | 0.05(-0.08,0.18) | 0.1( 0.00, 0.20) | 0.04(-0.05,0.12) | 0.09( 0.01, 0.16)* | -0.26(-0.83,0.31) | -0.61(-1.13,-0.10)* |

Notes: Adjusted by age, sex, race/ethnicity, educational attainment, marital status, BMI, smoking status, alcohol intake, milk product consumption, poverty income ratio, and serum cotinine. ^*^ Presenting for *P* < 0.05, ^**^ Presenting for *P* < 0.01, ^***^ Presenting for *P* < 0.001.

**Appendix Table 14** PRs (95%CIs) for the association between PFASs and periodontitis in the NHANES from 2009–2014 (Data without missing values in covariates) (N = 2736).

| Categories | Unadjusted model | | Adjusted model | |
| --- | --- | --- | --- | --- |
|  | PR (95%*CI*) | *P* value | PR (95%*CI*) | *P* value |
| PFOA [tertile (ng/mL)] |  |  |  |  |
| Low (＜1.87) | Ref | Ref | Ref | Ref |
| Middle (1.87-3.17) | 1.00(0.84,1.19) | 0.998 | 0.98(0.86,1.10) | 0.688 |
| High (≥3.17) | 0.95(0.76,1.20) | 0.685 | 1.01(0.89,1.14) | 0.909 |
| PFOS [tertile (ng/mL)] |  |  |  |  |
| Low (＜5.40） | Ref | Ref | Ref | Ref |
| Middle (5.40-10.90) | 1.07(0.81,1.41) | 0.646 | 1.03(0.87,1.22) | 0.734 |
| High (≥10.90） | 1.47(1.13,1.91) | **0.005** | 1.17(0.99,1.39) | 0.064 |
| PFHxS [tertile (ng/mL)] |  |  |  |  |
| Low (＜1.00） | Ref | Ref | Ref | Ref |
| Middle (1.00-2.07) | 1.22(1.02,1.46) | 0.031 | 0.96(0.85,1.08) | 0.506 |
| High (≥2.07） | 1.12(0.88,1.43) | 0.346 | 0.91(0.79,1.05) | 0.195 |
| MPAH [tertile (ng/mL)] |  |  |  |  |
| Low (＜0.07） | Ref | Ref | Ref | Ref |
| Middle (0.07-0.20) | 0.97(0.79,1.20) | 0.771 | 0.94(0.82,1.08) | 0.384 |
| High (≥0.20） | 1.25(0.99,1.58) | 0.061 | 1.08(0.92,1.26) | 0.328 |
| PFUA [tertile (ng/mL)] |  |  |  |  |
| Low (＜0.07） | Ref | Ref | Ref | Ref |
| Middle (0.07-0.20) | 0.93(0.71,1.23) | 0.620 | 1.05(0.88,1.24) | 0.600 |
| High (≥0.20） | 1.12(0.85,1.48) | 0.417 | 1.14(0.98,1.34) | 0.092 |
| PFNA [tertile (ng/mL)] |  |  |  |  |
| Low (＜0.74） | Ref | Ref | Ref | Ref |
| Middle (0.74-1.30) | 1.14(0.92,1.42) | 0.220 | 1.04(0.92,1.18) | 0.543 |
| High (≥1.30） | 1.36(1.05,1.77) | **0.022** | 1.17(1.01,1.35) | **0.037** |
| PFDE [tertile (ng/mL)] |  |  |  |  |
| Low (＜0.20) | Ref | Ref | Ref | Ref |
| Middle (0.20-0.30) | 1.06(0.84,1.35) | 0.608 | 1.04(0.89,1.22) | 0.595 |
| High (≥0.30） | 1.07(0.81,1.40) | 0.634 | 1.06(0.91,1.23) | 0.428 |

Notes: Ref = Reference. Adjusted by age, sex, race/ethnicity, educational attainment, marital status, BMI, smoking status, alcohol intake, milk product consumption, poverty income ratio, and serum cotinine.

**Appendix Table 15** E-values and lower limit of 95% CIs for the association between PFAS and periodontitis.

| Variables | E-value (lower limit of 95%CI) |
| --- | --- |
| PFOA [tertile (ng/mL)] |  |
| Low (＜1.87) | - |
| Middle (1.87-3.17) | 1.17(1.00) |
| High (≥3.17) | 1.14(1.00) |
| PFOS [tertile (ng/mL)] |  |
| Low (＜5.40） | - |
| Middle (5.40-10.90) | 1.18(1.00) |
| High (≥10.90） | 1.41(1.08) |
| PFHxS [tertile (ng/mL)] |  |
| Low (＜1.00） | - |
| Middle (1.00-2.07) | 1.00(1.00) |
| High (≥2.07） | 1.25(1.00) |
| MPAH [tertile (ng/mL)] |  |
| Low (＜0.07） | - |
| Middle (0.07-0.20) | 1.17(1.00) |
| High (≥0.20） | 1.26(1.00) |
| PFUA [tertile (ng/mL)] |  |
| Low (＜0.07） | - |
| Middle (0.07-0.20) | 1.11(1.00) |
| High (≥0.20） | 1.35(1.00) |
| PFNA [tertile (ng/mL)] |  |
| Low (＜0.74） | - |
| Middle (0.74-1.30) | 1.18(1.00) |
| High (≥1.30） | 1.38(1.11) |
| PFDE [tertile (ng/mL)] |  |
| Low (＜0.20) | - |
| Middle (0.20-0.30) | 1.24(1.00) |
| High (≥0.30） | 1.24(1.00) |

Notes: Ref = Reference. Adjusted by age, sex, race/ethnicity, educational attainment, marital status, BMI, smoking status, alcohol intake, milk product consumption, poverty income ratio, and serum cotinine.


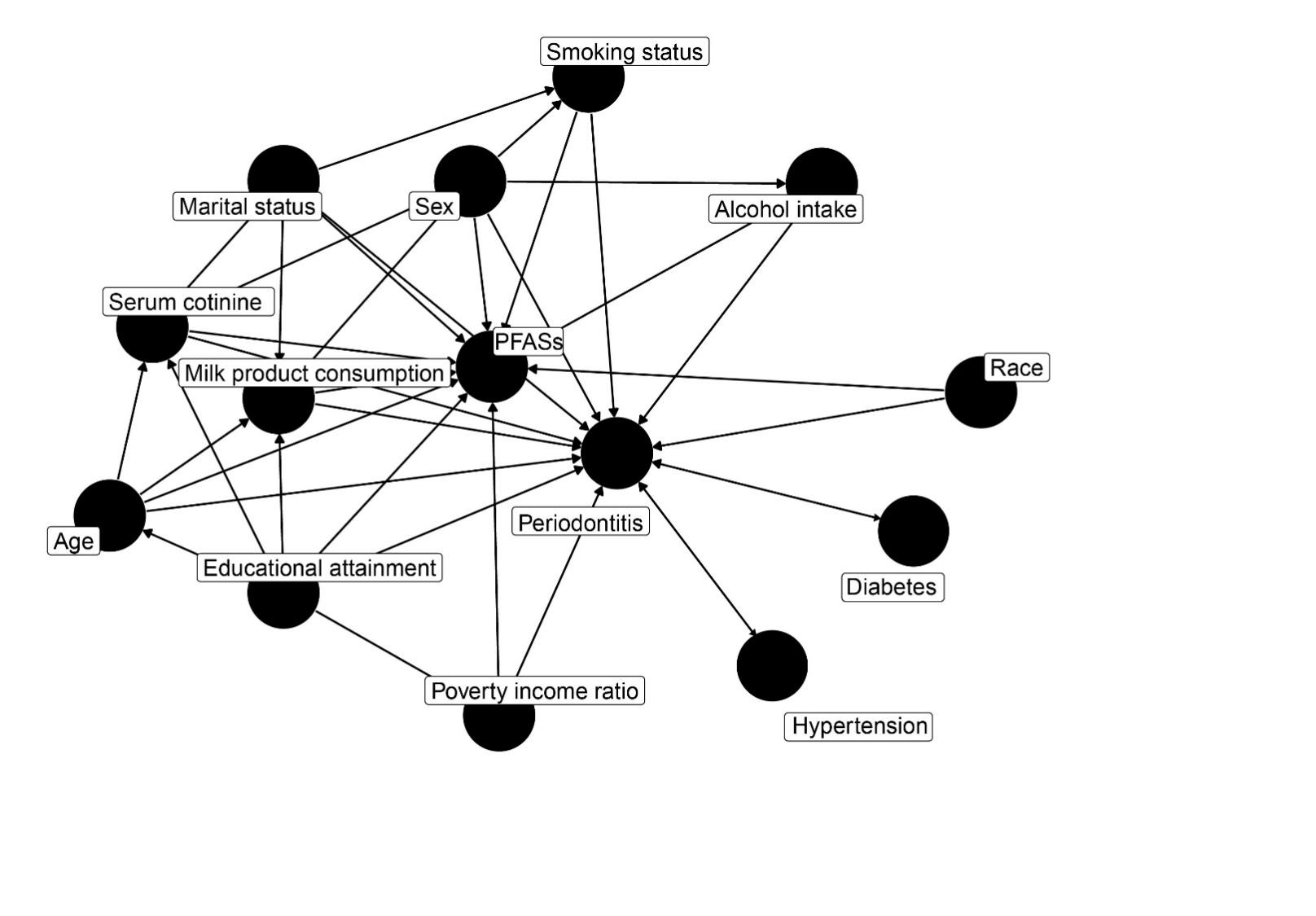


**Appendix Figure 1** Directed acyclic graph of confounding factors.

Notes: to PFASs: age[1], sex[2], educational attainment[3], marital status[4], race[5], poverty income ratio[4], smoking status[6], alcohol intake[7], serum cotinine [8], and milk product consumption[9].

To periodontitis: Age[10], sex[11], educational attainment[12], marital status[13], race[14], poverty income ratio[15], smoking status[11], alcohol intake[16], serum cotinine [17], milk product consumption [18], diabetes[19], hypertension[20, 21].


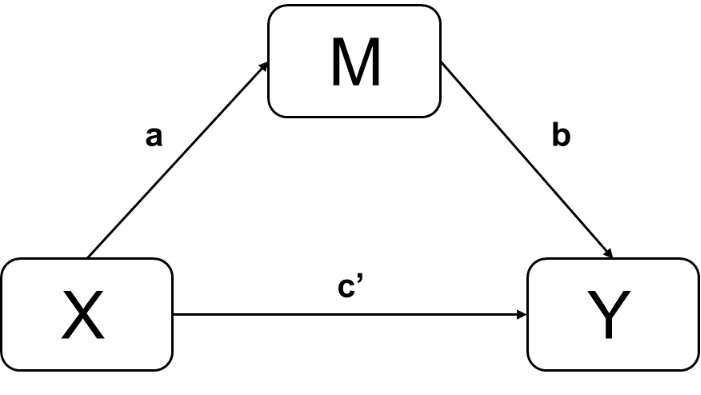


**Appendix Figure 2** Schematic of a simple mediation model.

Notes: The coefficient a represents the association between an independent variable (X) and a mediator variable (M), while the coefficient b signifies the relationship between M and a dependent variable (Y). Additionally, the coefficient c’ indicates the direct impact of X on Y. The indirect effect, denoted as ab, is computed by multiplying coefficients a and b. The total effect, which encompasses both direct and mediated effects, is represented by the sum of c’ and ab. The proportion mediated was determined by dividing the indirect effect (ab) by the total effect (c’ + ab).


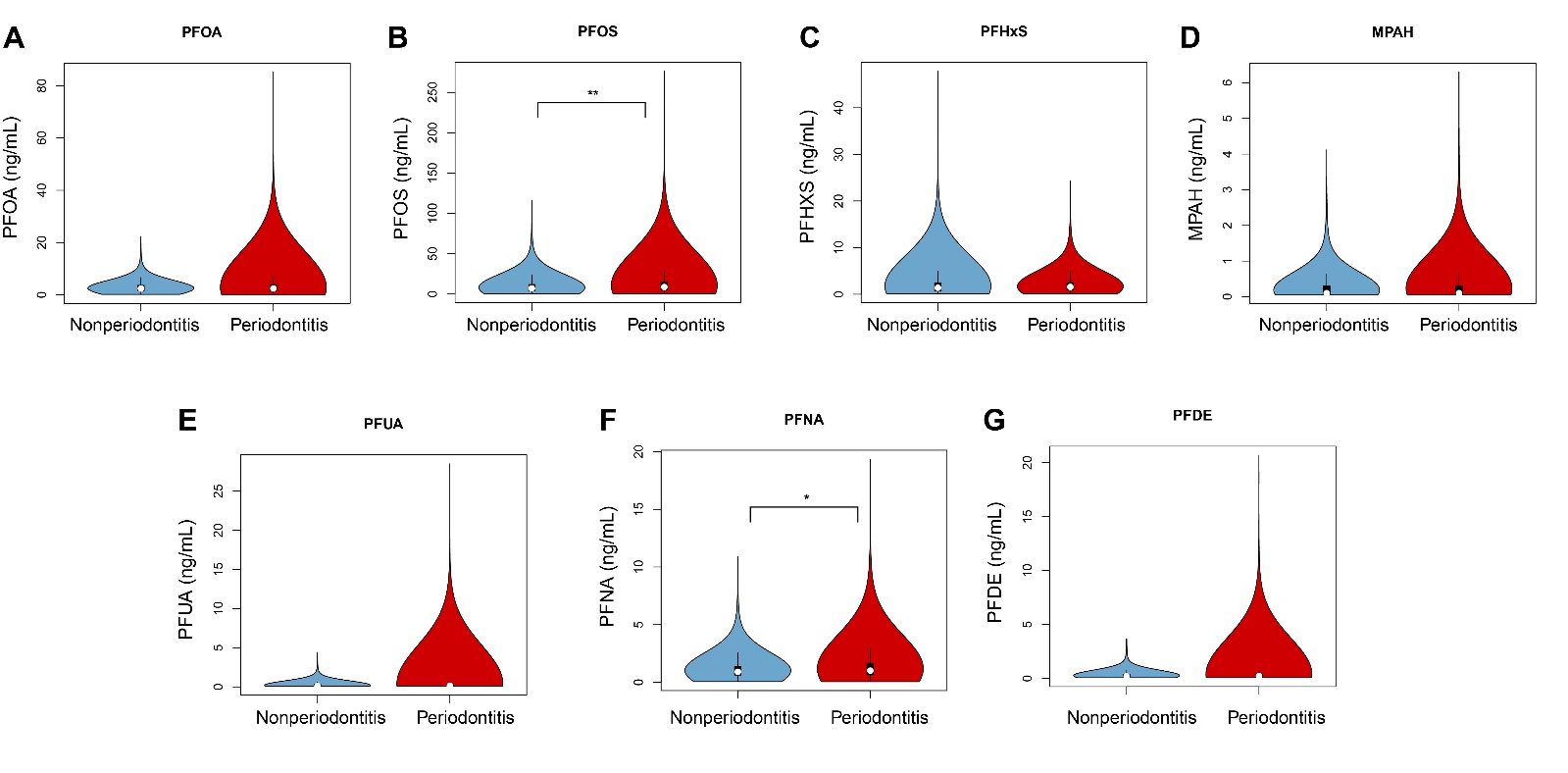


**Appendix Figure 3** Distribution of seven PFASs in NHANES participants (2009-2014).

Notes: NHANES, National Health and Nutrition Examination Survey; PFAS, per- and polyfluoroalkyl substances; PFOA, perfluorooctanoic acid; PFOS, perfluorooctane sulfonic acid; PFHxS, perfluorohexane sulfonate acid; MPAH, 2-(N-methyl-perfluorooctane sulfonamido) acetic acid; PFUA, perfluoroundecanoic acid; PFNA, perfluorononanoic acid; PFDE, perfluorodecanoic acid.


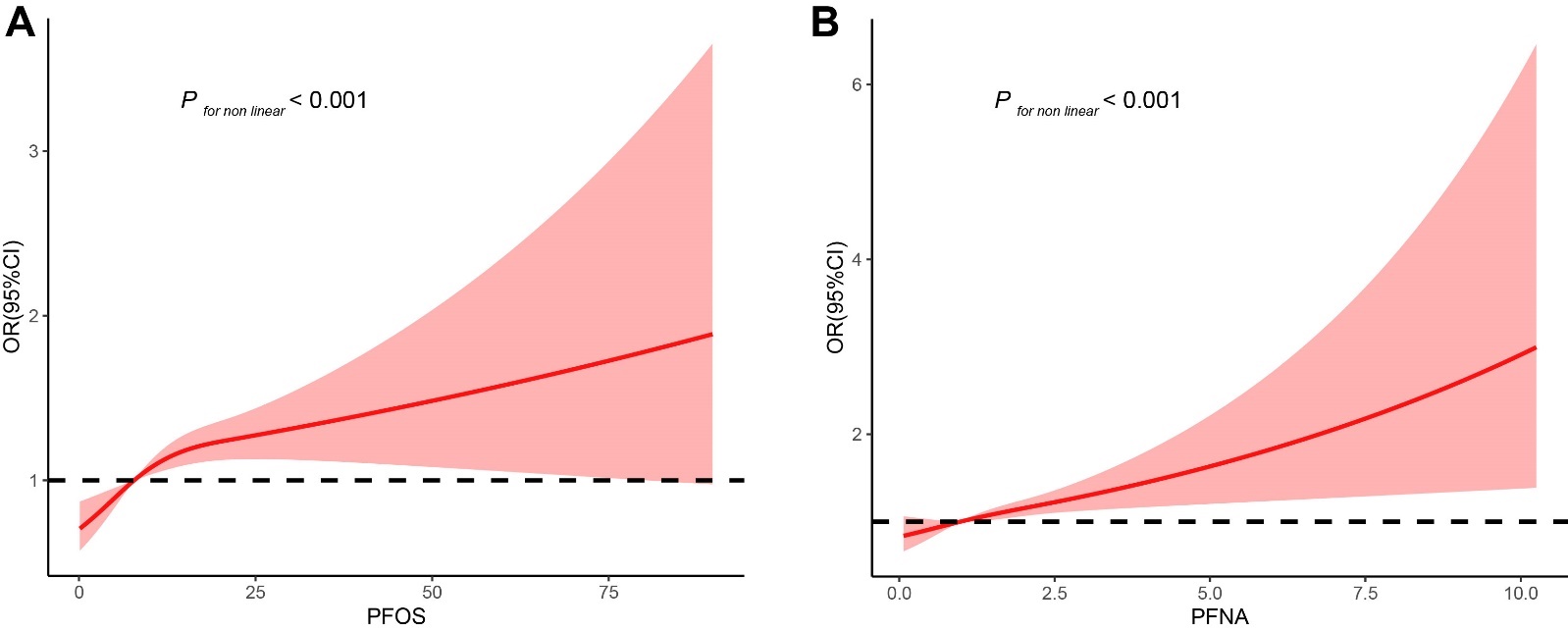


**Appendix Figure 4** Restricted cubic spline curves of serum PFOS/PFNA concentrations in NHANES 2009-2014 (N = 3248). Adjusted by age, sex, race/ethnicity, educational attainment, marital status, BMI, smoking status, alcohol intake, milk product consumption, poverty income ratio, and serum cotinine.


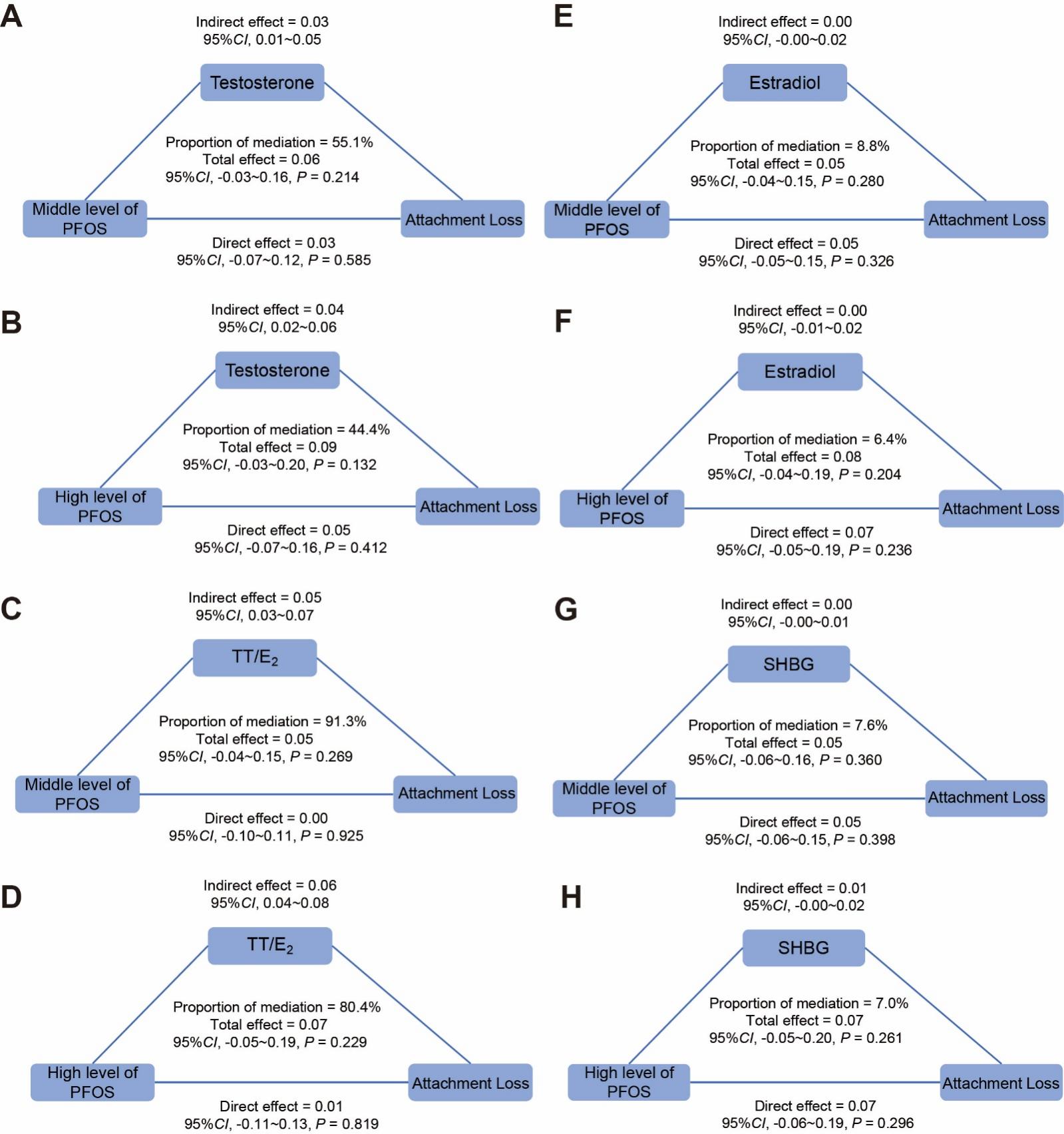


**Appendix Figure 5** Mediation analyses of association between PFOS and attachment loss (AL) by Testosterone (A, B), TT/E_2_ (a ratio of testosterone to estradiol) (C, D), Estradiol (E, F), SHBG (sex hormone binding protein) (G, H). Adjusted by age, race/ethnicity, educational attainment, marital status, BMI, smoking status, alcohol intake, milk product consumption, poverty income ratio, and serum cotinine.


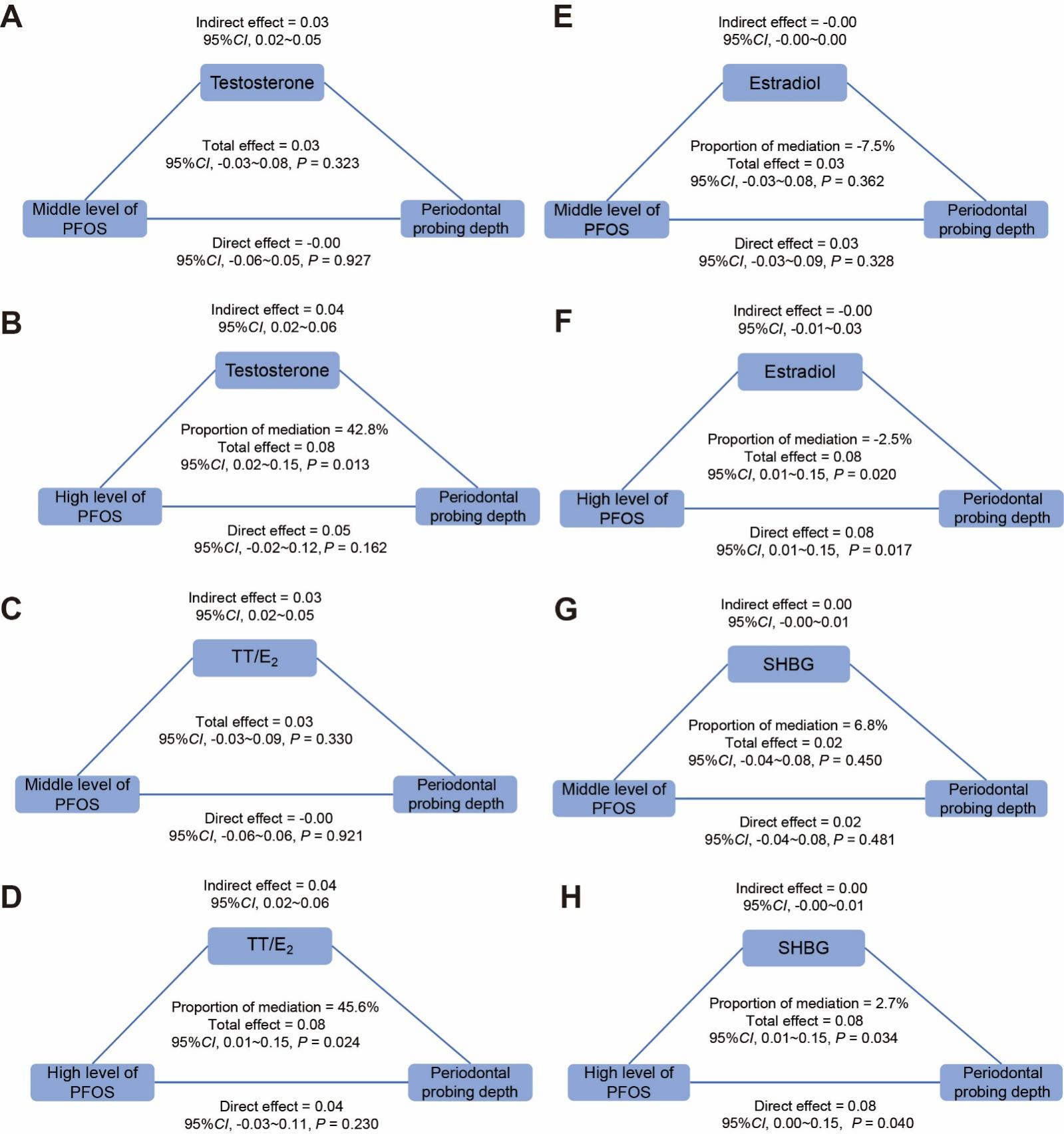


**Appendix Figure 6** Mediation analyses of association between PFOS and periodontal probing depth (PPD) by Testosterone (A, B), TT/E_2_ (a ratio of testosterone to estradiol) (C, D), Estradiol (E, F), SHBG (sex hormone binding protein) (G, H). Adjusted by age, race/ethnicity, educational attainment, marital status, BMI, smoking status, alcohol intake, milk product consumption, poverty income ratio, and serum cotinine.

**Reference**

1. Bulka CM, Avula V, Fry RC: **Associations of exposure to perfluoroalkyl substances individually and in mixtures with persistent infections: Recent findings from NHANES 1999-2016**. *Environ Pollut* 2021, **275**:116619.

2. Fenton SE, Ducatman A, Boobis A, DeWitt JC, Lau C, Ng C, Smith JS, Roberts SM: **Per- and Polyfluoroalkyl Substance Toxicity and Human Health Review: Current State of Knowledge and Strategies for Informing Future Research**. *Environ Toxicol Chem* 2021, **40**(3):606-630.

3. Kim K, Bennett DH, Calafat AM, Hertz-Picciotto I, Shin HM: **Temporal trends and determinants of serum concentrations of per- and polyfluoroalkyl substances among Northern California mothers with a young child, 2009-2016**. *Environ Res* 2020, **186**:109491.

4. DeLuca NM, Thomas K, Mullikin A, Slover R, Stanek LW, Pilant AN, Cohen Hubal EA: **Geographic and demographic variability in serum PFAS concentrations for pregnant women in the United States**. *J Expo Sci Environ Epidemiol* 2023, **33**(5):710-724.

5. Sagiv SK, Rifas-Shiman SL, Webster TF, Mora AM, Harris MH, Calafat AM, Ye X, Gillman MW, Oken E: **Sociodemographic and Perinatal Predictors of Early Pregnancy Per- and Polyfluoroalkyl Substance (PFAS) Concentrations**. *Environ Sci Technol* 2015, **49**(19):11849-11858.

6. Hall SM, Patton S, Petreas M, Zhang S, Phillips AL, Hoffman K, Stapleton HM: **Per- and Polyfluoroalkyl Substances in Dust Collected from Residential Homes and Fire Stations in North America**. *Environ Sci Technol* 2020, **54**(22):14558-14567.

7. Wise LA, Wesselink AK, Schildroth S, Calafat AM, Bethea TN, Geller RJ, Coleman CM, Fruh V, Claus Henn B, Botelho JC *et al*: **Correlates of plasma concentrations of per- and poly-fluoroalkyl substances among reproductive-aged Black women**. *Environ Res* 2022, **203**:111860.

8. McAdam J, Bell EM: **Determinants of maternal and neonatal PFAS concentrations: a review**. *Environ Health* 2023, **22**(1):41.

9. Hill NI, Becanova J, Lohmann R: **A sensitive method for the detection of legacy and emerging per- and polyfluorinated alkyl substances (PFAS) in dairy milk**. *Anal Bioanal Chem* 2022, **414**(3):1235-1243.

10. Campisi G, Chiappelli M, De Martinis M, Franco V, Ginaldi L, Guiglia R, Licastro F, Lio D: **Pathophysiology of age-related diseases**. *Immun Ageing* 2009, **6**:12.

11. Hong JW, Noh JH, Kim DJ: **The Prevalence and Associated Factors of Periodontitis According to Fasting Plasma Glucose in the Korean Adults: The 2012-2013 Korea National Health and Nutrition Examination Survey**. *Medicine (Baltimore)* 2016, **95**(14):e3226.

12. Borrell LN, Crawford ND: **Socioeconomic position indicators and periodontitis: examining the evidence**. *Periodontol 2000* 2012, **58**(1):69-83.

13. Obulareddy VT, Chava VK, Nagarakanti S: **Association of Stress, Salivary Cortisol, and Chronic Periodontitis: A Clinico-biochemical Study**. *Contemp Clin Dent* 2018, **9**(Suppl 2):S299-s304.

14. Boland MR, Hripcsak G, Albers DJ, Wei Y, Wilcox AB, Wei J, Li J, Lin S, Breene M, Myers R *et al*: **Discovering medical conditions associated with periodontitis using linked electronic health records**. *J Clin Periodontol* 2013, **40**(5):474-482.

15. Celeste RK, Oliveira SC, Junges R: **Threshold-effect of income on periodontitis and interactions with race/ethnicity and education**. *Rev Bras Epidemiol* 2019, **22**:e190001.

16. Arora PC, Ragi KGS, Arora A, Gupta A: **Oral Health Behavior and Treatment Needs among Drug Addicts and Controls in Amritsar District: A Case-controlled Study**. *J Neurosci Rural Pract* 2019, **10**(2):201-206.

17. Duque A, Martínez PJ, Giraldo A, Gualtero DF, Ardila CM, Contreras A, Duarte S, Lafaurie GI: **Accuracy of cotinine serum test to detect the smoking habit and its association with periodontal disease in a multicenter study**. *Med Oral Patol Oral Cir Bucal* 2017, **22**(4):e425-e431.

18. Adegboye AR, Christensen LB, Holm-Pedersen P, Avlund K, Boucher BJ, Heitmann BL: **Intake of dairy products in relation to periodontitis in older Danish adults**. *Nutrients* 2012, **4**(9):1219-1229.

19. Grossi SG, Genco RJ: **Periodontal disease and diabetes mellitus: a two-way relationship**. *Ann Periodontol* 1998, **3**(1):51-61.

20. Zhan Y, Jiao J, Jing W, Feng X, Tai B, Hu D, Lin HC, Wang B, Wang C, Zheng S *et al*: **Association between periodontitis and hypertension: cross-sectional survey from the Fourth National Oral Health Survey of China (2015-2016)**. *BMJ Open* 2023, **13**(3):e068724.

21. Kim HJ, Shim KW, Na HS, Kim SY, Yu Y, Song Y, Lee HA, Lee JY, Lee JH, Chung J: **Assessing the effect of antihypertensives on plaque microbiota in patients with periodontitis and hypertension using 16S rRNA sequencing: A cross-sectional study**. *J Periodontol* 2023, **94**(4):529-541.
